# Talaroacids A–D and Talaromarane A, Diterpenoids with Anti-Inflammatory Activities from Mangrove Endophytic Fungus *Talaromyces* sp. JNQQJ-4

**DOI:** 10.3390/ijms25126691

**Published:** 2024-06-18

**Authors:** Guisheng Wang, Jianying Wu, Zhaokun Li, Tao Chen, Yufeng Liu, Bo Wang, Yan Chen, Zhigang She

**Affiliations:** 1 School of Chemistry, Sun Yat-sen University, Guangzhou 510275, China; wanggsh9@mail2.sysu.edu.cn (G.W.); wujy89@mail2.sysu.edu.cn (J.W.); chent296@mail2.sysu.edu.cn (T.C.); liuyf76@mail2.sysu.edu.cn (Y.L.); ceswb@mail.sysu.edu.cn (B.W.); 2 School of Pharmacy, Anhui Medical University, Hefei 230032, China; lizhaokun0223@163.com

**Keywords:** mangrove endophytic fungus, diterpenoids, *Talaromyces* sp., anti-inflammatory

## Abstract

Five new diterpenes including four diterpenes with 1,2,3,4,4a,5,6,8a-octalin skeleton talaroacids A–D (**1**–**4**) and an isopimarane diterpenoid talaromarane A (**5**) were isolated from the mangrove endophytic fungus *Talaromyces* sp. JNQQJ-4. Their structures and absolute configurations were determined by analysis of high-resolution electrospray ionization mass spectroscopy (HRESIMS), 1D/2D Nuclear Magnetic Resonance (NMR) spectra, single-crystal X-ray diffraction, quantum chemical calculation, and electronic circular dichroism (ECD). Talaromarane A (**5**) contains a rare 2-oxabicyclo [3.2.1] octan moiety in isopimarane diterpenoids. In bioassays, compounds **1**, **2**, **4**, and **5** displayed significant anti-inflammatory activities with the IC_50_ value from 4.59 to 21.60 μM.

## 1. Introduction

Diterpenoids are a class of terpenoids containing 20 carbons and consist of four isopentenyl groups, which are widely distributed in animals, plants, and microorganisms [1]. More than 100 basic diterpene skeletons have been found, which can be divided into linear, monocarbocyclic, bicarbocyclic, tricarbocyclic, tetracarbocyclic, and pentacarbocyclic [2]. Among diverse skeleton types of diterpenes, diterpenes with decalin skeleton mainly include labdane [3], clerodane [4], and other types of diterpenes [5]. These diterpenes have a variety of pharmacological activities including anticancer [6,7], anti-inflammatory [8,9,10], antiparasitical [11], antiviral [12], enzyme inhibition [13,14], immunosuppressive [15], anti-angiogenesis [16], and antidiabetic [17].

**Figure 1:**
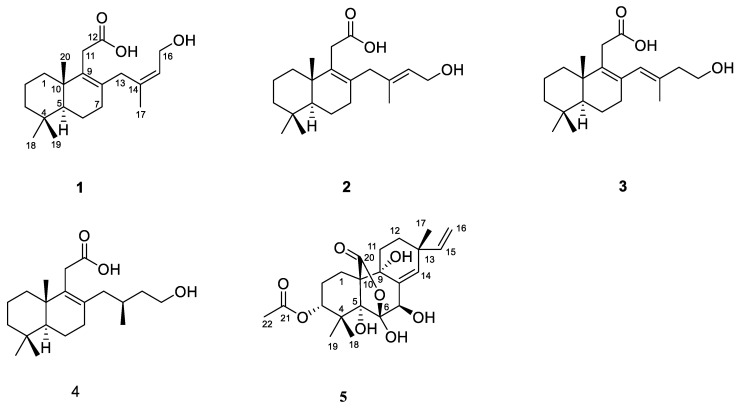
Structure of compounds **1**–**5**.

Mangrove ecosystems are usually located at the junction of land and ocean in tropical and subtropical regions and have abundant plant resources [18]. These mangrove plants, such as *Kandelia obovata*, were often used as traditional folk medicines [19]. In addition, due to the extreme environment of high salt, high temperature, local hypoxia, and periodic seawater immersion in mangroves, there are a variety of endophytic fungi resources [20]. Mangrove endophytic fungi can produce secondary metabolites with unique structures and remarkable biological activities, which capture the attention of numerous natural products and pharmacology researchers [21,22,23,24,25,26,27,28]. To date, more than 1300 new compounds have been identified from mangrove-derived fungi [29]. In our ongoing research search for bioactive compounds from mangrove endophytic fungi [30,31,32,33], the strain *Talaromyces* sp. JNQQJ-4 isolated from the leaf of *Kandelia obovata* was investigated. Four new diterpenes with 1,2,3,4,4a,5,6,8a-octalin skeleton talaroacids A–D (**1**–**4**) and a new isopimarane diterpenoid talaromarane A (**5**) were isolated from *Talaromyces* sp. JNQQJ-4 (Figure 1). In bioassay, compounds **1**, **2**, **4**, and **5** indicated significant anti-inflammatory activities with IC_50_ values from 4.59 to 21.60 µM. Herein, the isolation, structure elucidation, and biological assays of isolated diterpenoids are described.

## 2. Results and Discussion

### 2.1. Structure Identification

Talaroacid A (**1**) was obtained as a white powder, and had a molecular formula of C_20_H_32_O_3_ with five degrees of unsaturation based on the HRESIMS (Figure S1) data. The ^1^H NMR spectrum (Table 1 and Figure S2) indicated four methyl groups at *δ*_H_ 1.62 (s, H_3_-17), 0.95 (s, H_3_-20), 0.89 (s, H_3_-19), and 0.83 (s, H_3_-18); an olefinic proton signal at *δ*_H_ 5.49 (m, H-15). The ^13^C NMR (Table 2 and Figure S3) and HSQC spectra (Figure S4) data of **1** exhibited 20 carbon signals, including a carbonyl carbon, four methyls, four olefinic carbons (three non-hydrogenated carbons), eight methylenes (an oxygenated), a methine, and two quaternary carbons. 

**Figure 2:**
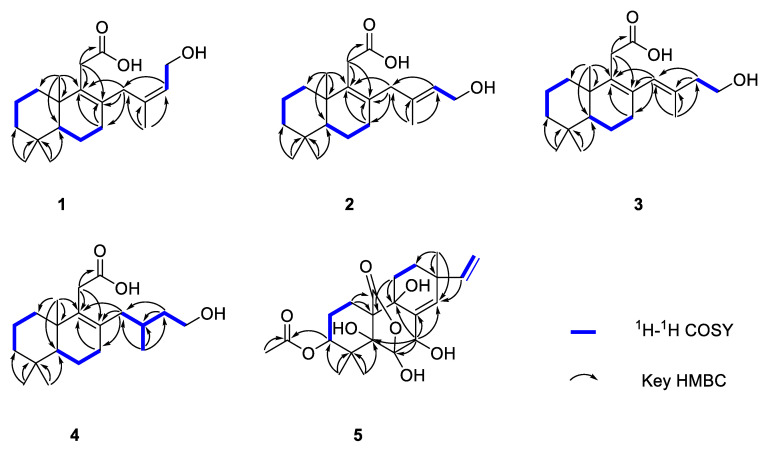
Key HMBC and COSY correlations of **1**–**5**.

**Figure 3:**
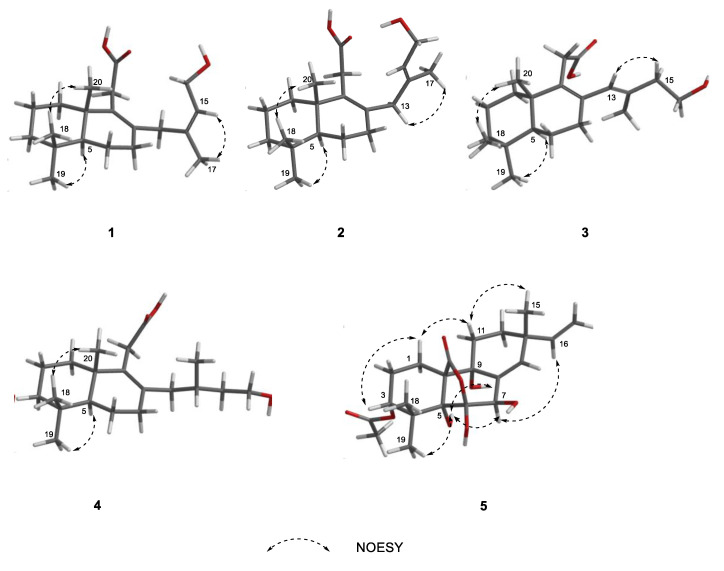
Key NOESY correlations of compounds **1**–**5**.

**Figure 4:**
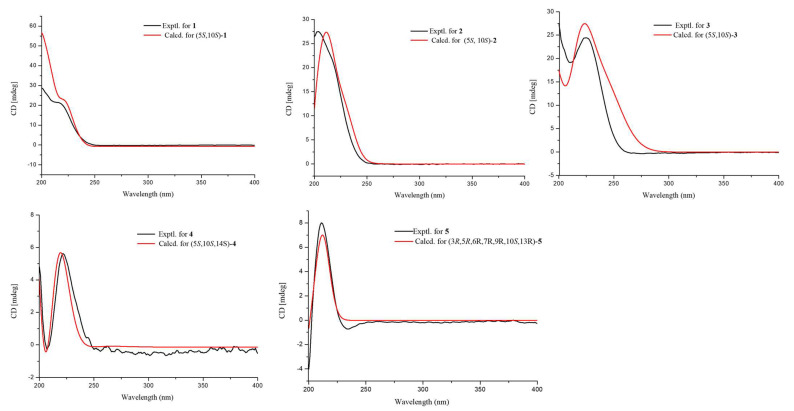
Experimental and calculated ECD spectra of compounds **1**–**5** in MeOH.

The key HMBC correlations (Figure 2 and Figure S6) from H_2_-7 to C-8 and C-9; from H_3_-18/H_3_-19 to C-3, C-4, and C-5; from H_3_-20 to C-1, C-5, C-9, and C-10 together with the spin system of H_2_-1/H_2_-2/H_2_-3 and H-5/H_2_-6/H_2_-7 indicated the presence of a 5,5,9-trimethyl-Δ^1,2^-octalin moiety of **1**. The HMBC correlations from H_2_-11 to C-8, C-9, C-10, and C-11 (*δ*_C_ 178.0) revealed the branched chain of acetic acid located at C-9. Furthermore, the spin coupling system (Figure S5) of H-15/H_2_-16 and the HMBC correlations from H_3_-17 to C-13, C-14, and C-15; from H_2_-16 to C-13; from H_2_-13 to C-7, C-8, and C-9 indicated the fragment of 3-methylbut-2-en-1-ol was linked to C-8. Thus, the planar structure of **1** was established and shown. The NOESY correlations (Figure 3 and Figure S7) between H-15 and H_3_-17 ensure the configuration of the Δ^14^ double bond as 14*Z*. Furthermore, the NOESY correlations of H_3_-18/H_3_-20 revealed they were positioned on the same face. In turn, the correlation of H-5/H_3_-19 suggested they were at the opposite orientation. Based on the above information, the relative configuration of **1** was assigned to 5S* and 10S*. Finally, the absolute configuration of **1** was determined as 5*S*, 10*S*, and *14Z* based on a comparison of experimental and calculated ECD spectra (Figure 4).

Talaroacid B (**2**) was obtained as a white powder. The HRESIMS data (Figure S8) suggested that **2** had the same molecular formula as that of **1**. The NMR data (Table 1 and Table 2; Figures S9–S11) closely resembled those of **1**, except for the chemical shift at C-15 (Δ*δ*_c_ −2.4). The ^1^H-^1^H COSY spectra and (Figure S12) and HMBC spectra (Figure S13) also indicate that compounds 2 and 1 have similar planar structures. The NOESY correlation (Figure 3 and Figure S14) of H-15/H_2_-13 indicated that the configuration of Δ^14^ double bond of **2** was 14*E*. Furthermore, the absolute configuration of **2** was determined as 5*S*, 10*S*, and *14E* according to the NOESY correlations and ECD calculation (Figure 4).

Talaroacid C (**3**) was obtained as a white powder and shared the same molecular formula as that of **2** based on the HRESIMS data (Figure S15). Comparing the NMR data (Table 1 and Table 2; Figures S16–S18) of compounds **3** and **2** showed that **3** had a similar structure to **2**. While, the ^1^H-^1^H COSY spectrum (Figure S19) and the HMBC correlations (Figure 2 and Figure S20) from H_2_-15 to C-13 (*δ*_C_ 129.2), C-14 (*δ*_C_ 135.1), and C-17 (*δ*_C_ 17.2); from H-13 (*δ*_H_ 5.65) to C-7 (*δ*_C_ 32.6), C-8 (*δ*_C_ 134.1), and C-9 (*δ*_C_ 137.6) revealed that the Δ^14^ double bond in **2** has changed to Δ^13^ double bond in **3**. Then, the configuration of Δ^13^ double bond of **3** was assigned as 13*E* based on the NOESY correlation of H-13/H_2_-15 (Figure 3 and Figure S21). Finally, the analysis of NOESY correlations and ECD calculation (Figure 4) determined the absolute configuration of **3** as 5*S*, 10*S*, and *13E*.

**Figure 5:**
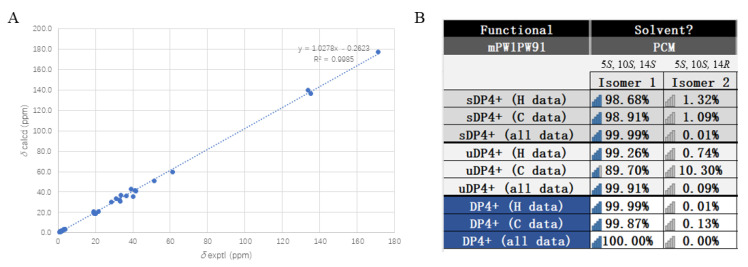
(**A**) Comparisons of calculated and experimental ^13^C NMR data of **4** (5*S*, 10*S,* 14*S*) in CDCl_3_; (**B**) DP4+ analysis of compound **4** including isomer **1** (5*S*, 10*S,* 14*S*) and isomer **2** (5*S*, 10*S,* 14*R*) in CDCl_3_.

Talaroacid D (**4**), a white powder, had a molecular formula of C_20_H_34_O_3_ and 4 degrees of unsaturation according to the HRESIMS data (Figure S22). The structure of **4** was similar to **2** by comparison of their NMR data (Table 1 and Table 2; Figures S23–S25). The main difference was the Δ^14^ double bond in **2** was reduced in **4**. The deduction was further confirmed by the ^1^H-^1^H correlations (Figure 2 and Figure S26) of H_2_-13/H-14(H_3_-17)/H_2_-15/H_2_-16 and the HMBC correlations (Figure 2 and Figure S27) from H_3_-17 to C-13 (*δ*_C_ 41.2), C-14 (*δ*_C_ 28.4), and C-15 (*δ*_C_ 40.1). Thus, the planar structure of **4** was established. According to the similar NOESY correlations (Figure 3 and Figure S28), the relative configuration of **4** was assigned to 5S* 10S*. Furthermore, to determine the relative configuration of C-14 in the side chain, the ^13^C NMR calculations of two possible structures (5S*,10S*,14S*)-**4** and (5S*,10S*,14R*)-**4** were performed using the gauge-including atomic orbital (GIAO) method at mPW1PW91-SCRF/6-311+G (d,p)/PCM (Chloroform). The results indicated that (5S*,10S*,14S*)-**4** was a reasonable structure (Figure 5, Figures S36 and S37) with a better correlation coefficient (R^2^ = 0.9985) and a high DP4+ probability score at 100% (all data). Finally, the absolute configuration of **4** was determined as 5*S*, 10*S*, and 14*S* based on the same experimental and calculated ECD spectra (Figure 4).

Talaromarane A (**5**) was obtained as a colorless crystal with the molecular formula of C_22_H_30_O_8_ and 8 degrees of unsaturation according to the HRESIMS data (Figure S29). The ^1^H NMR spectrum (Table 1 and Figure S30) revealed two hydroxyl proton signals at *δ*_H_ 4.94 (s, OH-5), and 3.90 (s, OH-7); four methyls at *δ*_H_ 2.13 (s, H_3_-22), 1.30 (s, H_3_-18), 1.27 (s, H_3_-19), and 1.00 (s, H_3_-17); four olefinic proton signals at *δ*_H_ 5.88 (s, H-14), 5.82 (dd, *J* = 17.5, 10.6 Hz, H-15), 5.04 (dd, *J* = 17.5, 1.0 Hz, H-16a), and 4.99 (dd, *J* = 10.6, 1.0 Hz, H-16b). Analysis ^13^C NMR (Table 2 and Figure S31) and HSQC data (Figure S32) to obtain 22 carbons including four methyls, five methylenes (one olefinic), four methines (two olefinic and an oxygenated), six non-hydrogenated carbons (two carbonyl carbons, three oxygenated and an olefinic), and three quaternary carbons. These data suggested that **5** belongs to an isopimarane diterpene [30]. The ^1^H-^1^H COSY correlations (Figure 2 and Figure S33) of H_2_-1/H_2_-2/H-3, H_2_-11/H_2_-12, and H-15/H_2_-16 together with the HMBC correlations from H-1 to C-10 and C-20; H_3_-18/19 to C-3, C-4 and C-5; H-7 to C-5, C-6, C-8, C-9, and C-14; H_3_-17 to C-12, C-13, and C-14; H-15 to C-14 and from H-11 to C-9 and C-10 to establish a typical tricyclic isopimarane diterpene skeleton. The acetyl group was located at C-3 based on the HMBC correlations (Figure 2 and Figure S34) from H_3_-22 (*δ*_H_ 2.13) and H-3 (*δ*_H_ 4.70) to C-21 (*δ*_C_ 168.9). The deshielding chemical shift at C-7 (*δ*_H_/*δ*_C_ 4.74/70.9) indicates that a hydroxyl group was located at C-7 in **5**. Moreover, the HMBC correlations from H-7 (*δ*_H_ 4.74) to non-hydrogenated carbons C-5 (*δ*_C_ 83.3), C-6 (*δ*_C_ 105.0), and C-9 (*δ*_C_ 73.2) revealed they were replaced by hydroxy groups, respectively. The HMBC correlations from H-1 to C-20 (*δ*_C_ 172.7) and the remaining unsaturation together with the deshielding chemical shift at non-hydrogenated carbon C-6 (*δ*_C_ 105.0) indicate that an oxygen bridge between C-7 and C-20. Thus, the plate structure of **5** was established.

**Figure 6:**
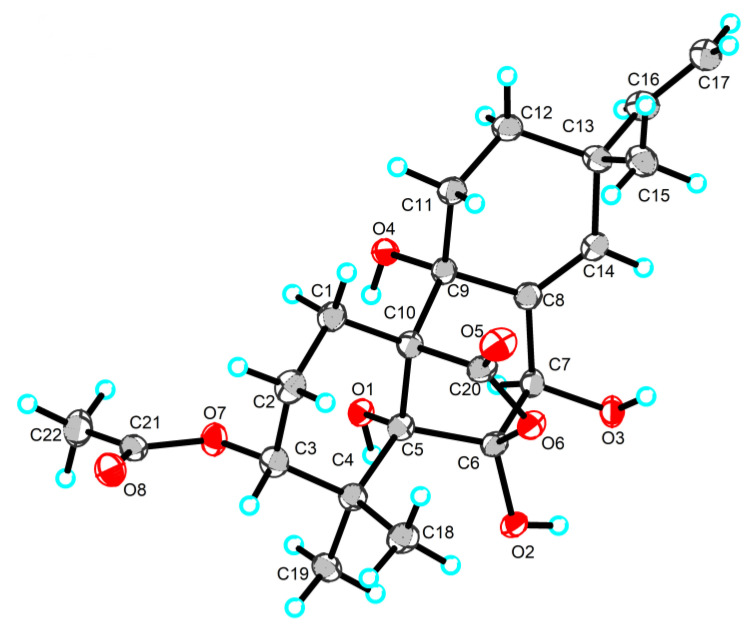
Single-crystal X-ray structures of compound **5**.

The NOESY correlations (Figure S35) of H-3/H-6/H-15/H_3_-18 indicated that these protons were in the same orientation. However, due to the absence of key NOE correlations for OH-5, OH-6, and OH-9 in the NOE spectrum (CDCl_3_), the relative configurations of **5** were difficult to determine. Luckily, the single crystal of **5** was successfully obtained by slow volatilization in MeOH. Finally, the absolute configuration of **5** was unambiguously determined as 3*R*, 5*R*, 6*R*, 7*R*, 9*R*, 10*S*, and 13*R* using single crystal X-ray diffraction analysis with a flack parameter of −0.22 (8) (Figure 6). In addition, ECD calculation also verifies the conclusion above (Figure 4).

### 2.2. Anti-Inflammatory Activities

On RAW264.7 cells test all compound’s cytotoxicity and anti-inflammatory activities (Table 3). The results indicated compound **2** had better anti-inflammatory activities than positive control quercetin (IC_50_ = 11.33 μM) with IC_50_ values of 4.59 μM. Compounds **1**, **4**, and **5** showed moderate anti-inflammatory activities with IC_50_ values of 15.78, 21.60, and 13.38 μM, respectively. None of the compounds were cytotoxic to RAW264.7 cells at the tested concentrations.

## 3. Materials and Methods

### 3.1. General Experiment Procedures

The optical rotations were recorded by using an MCP300 (Anton Paar, Shanghai, China). UV spectrum was obtained using a Shimadzu UV-2600 spectrophotometer (Shimadzu, Kyoto, Japan). The CD spectra were obtained from a J-810 spectropolarimeter in MeOH (JASCO, Tokyo, Japan). The IR data were performed on a Shimadzu IRTrace-100 spectrometer (Shimadzu, Tokyo, Japan) in KBr discs. All NMR data were measured on a Bruker Advance 600 MHz spectrometer at room temperature using the signals of residual solvent protons (CDCl_3_: *δ*_H_/*δ*_C_ 7.26/77.1; CD_3_OD: *δ*_H_/*δ*_C_ 3.31/49.2). The HRESIMS data were recorded by using a ThermoFisher LTQ-Orbitrap-LC-MS spectrometer (Palo Alto, CA, USA). Semi-preparative HPLC (Ultimate 3000 BioRS, Thermo Scientific, Waltham, MA, USA) was conducted using a semipreparative column (5 μm, 10 × 250 mm, Ultimate XB-C_18_, Welch Materials, Inc., Shanghai, China). A Rigaku XtaLAB Pro diffractometer (Rigaku, Tokyo, Japan) was used to obtain the crystallographic data of **5** (Cu Kα radiation). Column chromatography (CC) was performed using silica gel (200–300 mesh, Qingdao Marine Chemical, Qingdao, China) and Sephadex LH-20 (Sigma-aldrich, Saint Louis, MO, USA).

### 3.2. ECD and NMR Calculations

The ECD calculation was carried out using described previously [30]. The conformers were subjected to geometric optimization at the level of B3LYP/6-31+G (d,p) and the optimized conformers were calculated on the TD-DFT method using the B3LYP/6-311+G (d,p). All NMR calculations were performed using the GIAO method at mPW1PW91-SCRF/6-311+G (d,p)/PCM (Chloroform) [34].

### 3.3. Plant and Fungal Material

The healthy leaves of Kandelia obovata were collected in Jinniu Island Mangrove Nature Reserve in Guangzhou province of China in July 2023. The plant was identified by Dr. Yayue Liu, Guangdong Ocean University, and voucher sp. (JNQJ202306) is stored at Sun Yat-sen University. The strain *Talaromyces* sp. JNQQJ-4 was isolated from the healthy leaves of *Kandelia obovate*. The specific separation process was as follows: the fresh leaf tissue of *Kandelia candel* was transferred to 3% sodium hypochlorite solution and 75% ethanol solution with sterilized tweezers, and washed with sterile water. The leaf tissue was cut into regular small pieces (about 0.2 × 0.6 cm) and cultured on an autoclaved Bengal Rose agar plate incubated at 28 °C for 3 days. After the colony appeared, the mycelia were picked and inoculated on PDA medium. Repeat the above steps until a pure single colony is obtained on PDA plate. Fungal species were identified using DNA amplification and ITS sequence analysis previously [35]. The strain sequence data were reserved for the GenBank with accession number PP660349, and BLAST analysis revealed that it was 100% homologous to the sequence of *Talaromyces* sp. (MK450749.1). This strain was preserved at Sun Yat-sen University, China.

### 3.4. Fermentation, Extraction and Purification

The fungal strain was seeded to sixty 1 L Erlenmeyer flasks with 70 g raw rice and 30 mL 0.3% seawater and incubated at 25 °C for 28 days. The solid rice media were extracted with ethyl acetate and concentrated to obtain 45.9 g of crude extract. Five fractions (Fr.A-Fr.E) were isolated from the extract using silica gel CC (200–300 mesh) eluting with petroleum ether/ethyl acetate gradient (1:0~0:1). Fractions B was purified using CC on silica gel (CH_2_Cl_2_/MeOH, 80:1) and Sephadex LH-20 (CH_2_Cl_2_/MeOH, 1:1) to produce subfractions B_1_–B_3_. Fr. B_2_ was purified using semipreparative HPLC (CH_3_CN/H_2_O/Trifluoroacetic acid, 60:40:0.05, 1.5 mL/min) to obtain compounds **1** (5.3 mg, t_R_ = 13.5 min), **2** (4.8 mg, t_R_ = 14.5 min), **3** (4.5 mg, t_R_ = 17.0 min), and **4** (3.6 mg, t_R_ = 19.0 min). Fr. C_1_–C_4_ was obtained by separating fractions C using CC on a silica gel (CH_2_Cl_2_/MeOH, 75:1). Then, compound **5** (3.3 mg, t_R_ = 14.2 min) was obtained using semipreparative HPLC (CH_3_CN /H_2_O, 70:30, 1.5 mL/min) from Fr. C_1_.

Talaroacid A (**1**): white powder.; ${\left[\alpha \right]}_{D}^{25}$ = 10.4 (*c* 0.23, MeOH); UV (MeOH) *λ*_max_ (log *ε*): 201 (2.26) nm; ECD (*c* 0.33 mM, MeOH) *λ*_max_ (Δ*ε*) 201 (30.5), 218 (20.3) nm; IR (KBr) *ν*_max_: 3326, 2935, 1706, 1445, 1385 and 1180 cm^−1^; ^1^H NMR (500 MHz, MeOH-*d*_4_) data, Table 1; ^13^C NMR (125 MHz, MeOH-*d*_4_) data, Table 2; HRESIMS *m*/*z* 343.2246 [M + Na]^+^ (calcd: C_20_H_32_O_3_Na, 343.2244).

Talaroacid B (**2**): white powder.; ${\left[\alpha \right]}_{D}^{25}$ = 8.8 (*c* 0.25, MeOH); UV (MeOH) *λ*_max_ (log *ε*): 201 (1.56) nm; ECD (*c* 0.33 mM, MeOH) *λ*_max_ (Δ*ε*) 203 (27.5) nm; IR (KBr) *ν*_max_: 3328, 2940, 1712, 1447, 1390 and 1175 cm^−1^; ^1^H NMR (500 MHz, CDCl_3_) data, Table 1; ^13^C NMR (125 MHz, CDCl_3_) data, Table 2; HRESIMS *m*/*z* 343.2238 [M + Na]^+^ (calcd: C_20_H_32_O_3_Na, 343.2244).

Talaroacid C (**3**): white powder.; ${\left[\alpha \right]}_{D}^{25}$ = 12.3 (*c* 0.28, MeOH); UV (MeOH) *λ*_max_ (log *ε*): 201 (1.88) nm; ECD (*c* 0.33 mM, MeOH) *λ*_max_ (Δ*ε*) 208 (19.5), 225 (25.3) nm; IR (KBr) *ν*_max_: 3327, 2938, 1716, 1450, 1388 and 1171 cm^−1^; ^1^H NMR (500 MHz, CDCl_3_) data, Table 1; ^13^C NMR (125 MHz, MeOH-*d*_4_) data, Table 2; HRESIMS *m*/*z* 343.2246 [M + Na]^+^ (calcd: C_20_H_32_O_3_Na, 343.2244).

Talaroacid D (**4**): white powder.; ${\left[\alpha \right]}_{D}^{25}$ = 5.3 (*c* 0.28, MeOH); UV (MeOH) *λ*_max_ (log *ε*): 201 (1.23) nm; ECD (*c* 0.33 mM, MeOH) *λ*_max_ (Δ*ε*) 201 (1.5), 225 (1.8) nm; IR (KBr) *ν*_max_: 3325, 2936, 1714, 1448, 1389 and 1173 cm^−1^; ^1^H NMR (500 MHz, CDCl_3_) data, Table 1; ^13^C NMR (125 MHz, CDCl_3_) data, Table 2; HRESIMS *m*/*z* 345.2401 [M + Na]^+^ (calcd: C_20_H_34_O_3_Na, 345.2400).

Talaromarane A (**5**): colorless crystal; ${\left[\alpha \right]}_{D}^{25}$ = 8.3 (*c* 0.30, MeOH); UV (MeOH) *λ*_max_ (log *ε*): 201 (1.80) nm; ECD (*c* 0.35 mM, MeOH) *λ*_max_ (Δ*ε*) 210 (8.0); IR (KBr) *ν*_max_: 3422, 2928, 1637 cm^−1^; ^1^H NMR (500 MHz, CDCl_3_) data, Table 1; ^13^C NMR (125 MHz, CDCl_3_) data, Table 2; HRESIMS *m*/*z* 421.1869 [M − H]^−^ (calcd: C_22_H_29_O_7_, 421.1868).

### 3.5. Crystallographic Data for Talaromarane A

The X-ray diffraction data of talaromarane A (**5**) were measured using a Rigaku XtaLAB Pro diffractometer with CuKα radiation (λ = 1.54184 Å). The structure of **5** was resolved using SHELXT methods and refined by full-matrix least-squares difference Fourier techniques on an OLEX2 interface program. The crystallographic data of **5** were preserved at the Cambridge Crystallographic Data Centre.

Molecular formula C_22_H_30_O_8_, formula weight 422.46, orthorhombic, space group = P2_1_2_1_2_1_, unit cell: a = 8.71630 (10) Å α = 90°, b = 11.06960(10) Å β = 90°, c = 21.1646(2) Å γ = 90°, V = 2042.09(4) Å^3^, ρ_calcg_ = 1.374 cm^3^, Z = 4, T = 99.98(10) K, μ (CuKα) = 0.868 mm^−1^, F (000) = 904.0. A total of 16096 reflections (8.356° ≤ 2Θ ≤ 148.688°) were measured with 4108 independent reflections (R_int_ = 0.0453, R_sigma_ = 0.0337). Final R indexes [I ≥ 2σ (I)]: R_1_ = 0.0320, wR_2_ = 0.0841. Final R indexes [all data]: R_1_ = 0.0336, wR_2_ = 0.0841. Largest diff. peak and hole = 0.24 and −0.18 eÅ^−3^. Flack parameter = −0.22 (8). Crystallographic data for the structure reported in this paper were deposited in the Cambridge Crystallographic Data Centre (Accession No. CCDC 2351536).

### 3.6. Anti-Inflammatory Assay

Standard Anti-inflammatory assays employing RAW264.7 cell lines were carried out as described previously [30]. All compounds were tested for cytotoxic activity before anti-inflammatory testing. The RAW264.7 cells were cultured in Dulbecco’s modified Eagle’s medium (DMEM, Gibco, NY, USA) at 37 °C with 5% CO_2_ humidified incubator. Quercetin (Sigma, Burlington, VT, USA) or compound was dissolved in DMSO to prepare mother liquor (10 mm/mL). Cytotoxic activity was tested by MTT assay. The cells were pretreated with different concentrations of quercetin or compounds (5, 10, 20, 30, 40, and 50 µM) for 24 h, then 10 µL of MTT (0.5 mg/mL) was added to each well and cultured for 4 h to test the absorbance at 540 nm. The concentration of DMSO was 0.2% of the medium culture. The NO content was determined by the Griess method to evaluate the anti-inflammatory activity of the compounds. Firstly, 500 μL cells (3 × 10^6^ cells/mL) were seeded in 24-well plates and cultured overnight. Different concentrations of quercetin or compounds (5, 10, 20, 30, 40, and 50 µM) pretreated with LPS were added and cultured for 24 h, and the absorbance of final products was measured at 540 nm. None compounds displayed cytotoxic on RAW264.7 cell at 50 µM. Quercetin was the positive control.

### 3.7. Solubility and the Stability

Compounds **1**–**5** were dissolved in chloroform, and no change in compounds **1**–**5** was found by TLC detection after overnight storage. It was shown that compounds **1**–**5** were stable under normal conditions.

## 4. Conclusions

In conclusion, four new diterpenes with 1,2,3,4,4a,5,6,8a-octalin skeleton talaroacids A-D (**1**–**4**) and a new isopimarane diterpenoid talaromarane A (**5**) were isolated from the mangrove endophytic fungus *Talaromyces* sp. JNQQJ-4. It is noteworthy that **5** contains a rare 2-oxabicyclo [3.2.1] octan moiety in isopimarane. Moreover, compound **2** exhibited promising NO inhibitory activity with IC_50_ values of 4.59 μM. In addition, the better activity of compounds **1**–**2** than **3**–**4** indicated that the Δ^14^ double bond in the side chain makes a contribution to NO inhibitory activity. Nitric oxide (NO) is a signaling molecule produced by inducible nitric oxide synthase (iNOS), playing an important regulatory role in the occurrence and development of inflammation [36]. It is closely related to many major inflammation-induced diseases, such as autoimmune diseases, arthritis, cardiovascular diseases, and diabetes [37]. Inhibiting the production of NO can reduce inflammatory responses and prevent subsequent diseases [38]. Therefore, NO inhibitors were considered a promising direction for anti-inflammatory drug research [39]. Recently, several diterpenes with decalin skeleton have been reported to have significant NO inhibitory activity [40,41,42]. Among diterpenes, tinopanoid M, a clerodane diterpenoid isolated from *Tinospora crispa,* exerts good anti-inflammatory effects by reducing the expression of various pro-inflammatory factors and modulating multiple inflammatory pathways [41]. Thus, talaroacid B (**2**) might be worthy of further study as a potential anti-inflammatory lead compound.

## Figures and Tables

**Table 1:** ^1^H NMR (600 MHz) data of compounds **1**–**5** (*δ*_H_ in ppm, *J* in Hz).

No.	1 *^b^*	2 *^a^*	3 *^b^*	4 *^a^*	5 *^a^*
1a	1.67, m	1.68, m	1.73, m	1.68, m	2.05, m
1b	1.20, overlap	1.22, overlap	1.26, m	1.19, m	
2a	1.65, m	1.45, m	1.64, m	1.69, m	1.91, m
2b	1.39, overlap	1.21, overlap	1.48, overlap	1.21, m	
3a	1.38, m	1.39, m	1.43, m	1.38, m	4.70, dd (3.5, 2.2)
3b	1.15, m	1.16, m	1.19, m	1.15, m	
5	1.18, m	1.22, overlap	1.27, overlap	1.20, overlap	
6a	1.57, m	1.58, m	1.74, overlap	1.58, m	
6b	1.47, m	1.27, m	1.27, overlap	1.45, m	
7	1.99, m	2.04, m	2.05, m	2.08, m	4.74, s
11a	3.23, d (17.4)	3.10, d (17.3)	3.03, d (16.7)	3.20, d (17.4)	1.76, m
11b	3.04, d (17.4)	2.99, d (17.3)	2.90, d (16.7)	3.00, d (17.4)	1.69, m
12a					1.96, m
12b					1.49, m
13a	2.84, d (14.6)	2.78, d (16.0)	5.65, s	1.88, d (7.5)	
13b	2.71, d (14.6)	2.54 d (16.0)			
14				1.82, m	5.88, s
15a	5.49, m	5.32, t (7.1)	2.23, t (7.1)	1.59, m	5.82, dd (17.5, 10.6)
15b				1.36, m	
16a	4.18, m	4.15, d (7.1)	3.62, t (7.1)	3.68, m	5.04, dd (17.5, 1.0)
16b					4.99, dd (10.6, 1.0)
17	1.62, s	1.62, s	1.56, s	0.84, overlap	1.00, s
18	0.83, s	0.84, s	0.87, s	0.83, s	1.27, s
19	0.89, s	0.89, s	0.91, s	0.89, s	1.30, s
20	0.95, s	0.98, s	1.02, s	0.95, s	
22					2.13, s

*^a^* Measured in CDCl_3_, *^b^* Measured in MeOH-*d*_4_.

**Table 2:** ^13^C NMR (150 MHz) data of compounds **1**–**5** (*δ*_C_ in ppm).

No.	1 *^b^*	2 *^a^*	3 *^b^*	4 *^a^*	5 *^a^*
1	36.3, CH_2_	36.2, CH_2_,	37.4, CH_2_	36.4, CH_2_,	14.4, CH_2_
2	18.9, CH_2_	18.9, CH_2_	19.9, CH_2_	19.1, CH_2_	21.8, CH_2_
3	41.6, CH_2_	41.6, CH_2_	42.9, CH_2_	41.6, CH_2_	80.2, CH
4	33.4, C	33.5, C	34.2, C	33.5, C	40.8, C
5	51.4, CH	51.4, CH	52.7, CH	51.4, CH	83.3, C
6	19.0, CH_2_	19.0 *^c^*, CH_2_	20.0, CH_2_	19.0, CH_2_	105.0, C
7	31.1, CH_2_	31.9, CH_2_	32.6, CH_2_	31.1, CH_2_	70.9, CH
8	132.1, C	132.0, C	134.1, C	133.6, C	135.4, C
9	135.8, C	136.7, C	137.6, C	135.2, C	73.2, C
10	39.1, C	39.1, C	39.7, C	39.1, C	55.9, C
11	32.7, CH_2_	32.7, CH_2_	34.7, CH_2_	32.8, CH_2_	27.2, CH_2_
12	178.0, C	177.0, C	176.9, C	171.2, C	29.6, CH_2_
13	35.9, CH_2_	43.3, CH_2_	129.2, CH	41.2, CH_2_	38.3, C
14	138.3, C	137.8, C	135.1, C	28.4, CH	135.9, CH
15	125.6, CH	123.2, CH	43.0, CH_2_	40.1, CH_2_	146.9, CH
16	59.1, CH_2_	59.5, CH_2_	61.8, CH_2_	61.2, CH_2_	111.7, CH_2_
17	22.9, CH_3_	16.8, CH_3_	17.2, CH_3_	19.4, CH_3_	24.3, CH_3_
18	21.8, CH_3_	21.8, CH_3_	22.1, CH_3_	21.8, CH_3_	24.4, CH_3_
19	33.3, CH_3_	33.3, CH_3_	33.7, CH_3_	33.3, CH_3_	22.2, CH_3_
20	19.9, CH_3_	20.2, CH_3_	20.4, CH_3_	20.2, CH_3_	172.7, C
21					168.9, C
22					21.4, CH_3_

*^a^* Measured in CDCl_3_, *^b^* Measured in MeOH-*d*_4_, *^c^* overlap in ^13^C NMR.

**Table 3:** Inhibitory Effects against NO Production of Compounds **1**–**5** in LPS-Induced RAW264.7 Cells.

Compounds	1	2	3	4	5	Quercetin
IC50 (μM)	15.78	4.59	>50	21.60	13.38	11.33

## Data Availability

Data is contained within the article and Supplementary Materials.

## Supplementary Materials

The following are available online at https://www.mdpi.com/article/10.3390/ijms25126691/s1.
