# Supplementary material for: Talaroacids A–D and Talaromarane A, Diterpenoids with Anti-Inflammatory Activities from Mangrove Endophytic Fungus Talaromyces sp. JNQQJ-4
Source: Int J Mol Sci. 2024 Jun 18;25(12):6691. doi: 10.3390/ijms25126691 (PMC11204306; doi:10.3390/ijms25126691)
Supplement: Supplementary file 1 [file ijms-25-06691-s001.zip › ijms-3050304-supplementary.pdf]

Supplementary Material

Talaroacids A–D and Talaromarane A, Diterpenoids with Anti-Inflammatory Activities from Mangrove Endophytic Fungus *Talaromyces* sp. JNQQJ-4

Guisheng Wang [1], Jianying Wu [1], Zhaokun Li [2], Tao Chen [1], Yufeng Liu [1], Bo Wang [1], Yan Chen [2,*] and Zhigang She [1,*]

[1] School of Chemistry, Sun Yat-sen University, Guangzhou 510275, China; wanggsh9@mail2.sysu.edu.cn (G.W.); wujy89@mail2.sysu.edu.cn (J.W.); chent296@mail2.sysu.edu.cn (T.C.); liuyf76@mail2.sysu.edu.cn (Y.L.); ceswb@mail.sysu.edu.cn (B.W.)

[2] School of Pharmacy, Anhui Medical University, Hefei 230032, China; lizhaokun0223@163.com

* Correspondence: 2022500051@ahmu.edu.cn (Y.C.); esshzhg@mail.sysu.edu.cn (Z.S.)

Figure S1. HRESIMS spectrum of **1**
Figure S2. ^{1}H-NMR spectrum of **1** in CD_3OD
Figure S3. ^{13}C-NMR spectrum of **1** in CD_3OD
Figure S4. HSQC spectrum of **1**
Figure S5. ^{1}H-^{1}H COSY spectrum of **1**
Figure S6. HMBC spectrum of **1**
Figure S7. NOESY spectrum of **1**
Figure S8. HRESIMS spectrum of **2**
Figure S9. ^{1}H-NMR spectrum of **2** in $CDCl_3$
Figure S10. ^{13}C-NMR spectrum of **2** in $CDCl_3$
Figure S11. HSQC spectrum of **2**
Figure S12. ^{1}H-^{1}H COSY spectrum of **2**
Figure S13. HMBC spectrum of **2**
Figure S14. NOESY spectrum of **2**
Figure S15. HRESIMS spectrum of **3**
Figure S16. ^{1}H-NMR spectrum of **3** in CD_3OD
Figure S17. ^{13}C-NMR spectrum of **3** in CD_3OD
Figure S18. HSQC spectrum of **3**
Figure S19. ^{1}H-^{1}H COSY spectrum of **3**
Figure S20. HMBC spectrum of **3**
Figure S21. NOESY spectrum of **3**
Figure S22. HRESIMS spectrum of **4**
Figure S23. ^{1}H-NMR spectrum of **4** in $CDCl_3$
Figure S24. ^{13}C-NMR spectrum of **4** in $CDCl_3$
Figure S25. HSQC spectrum of **4**
Figure S26. ^{1}H-^{1}H COSY spectrum of **4**
Figure S27. HMBC spectrum of **4**
Figure S28. NOESY spectrum of **4**
Figure S29. HRESIMS spectrum of **5**
Figure S30. ^{1}H-NMR spectrum of **5** in $CDCl_3$
Figure S31. ^{13}C-NMR spectrum of **5** in $CDCl_3$
Figure S32. HSQC spectrum of **5**
Figure S33. ^{1}H-^{1}H COSY spectrum of **5**
Figure S34. HMBC spectrum of **5**
Figure S35. NOESY spectrum of **5**
Figure S36. Comparison of the experimental ^{13}C and ^{1}H NMR data of compound **4** and calculated chemical shifts for two potential epimers (5*S*, 10*S*, 14*S* and 5*S*, 10*S*, 14*R*)
Figure S37. DP4+ analysis of **4** (isomer1 = 5*S*, 10*S*, 14*S* and isomer1 = 5*S*, 10*S*, 14*R*)

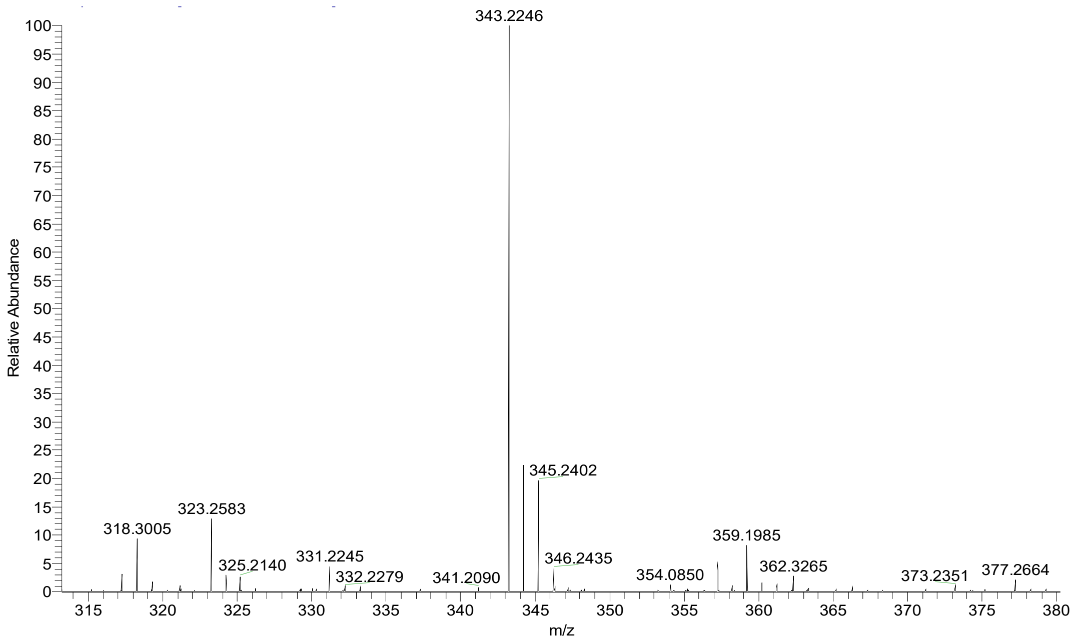

Figure S1. HRESIMS spectrum of compound **1**

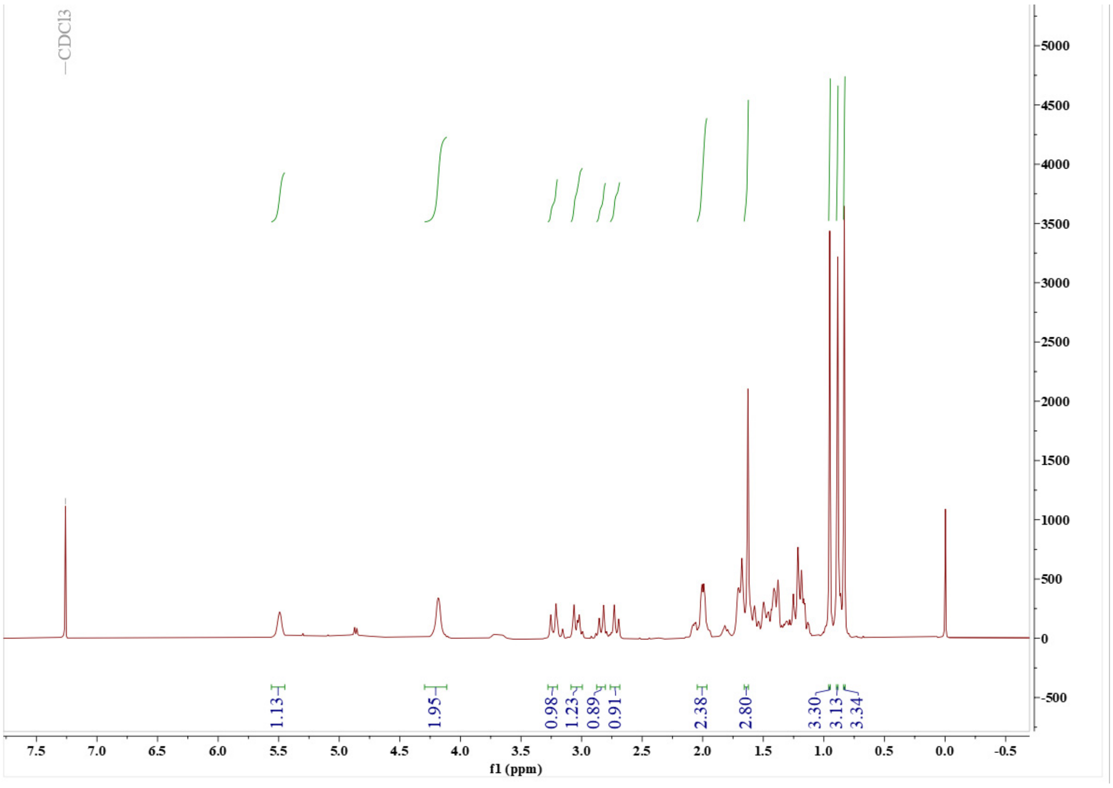

Figure S2. ^{1}H NMR spectrum of compound **1** in CD_3OD

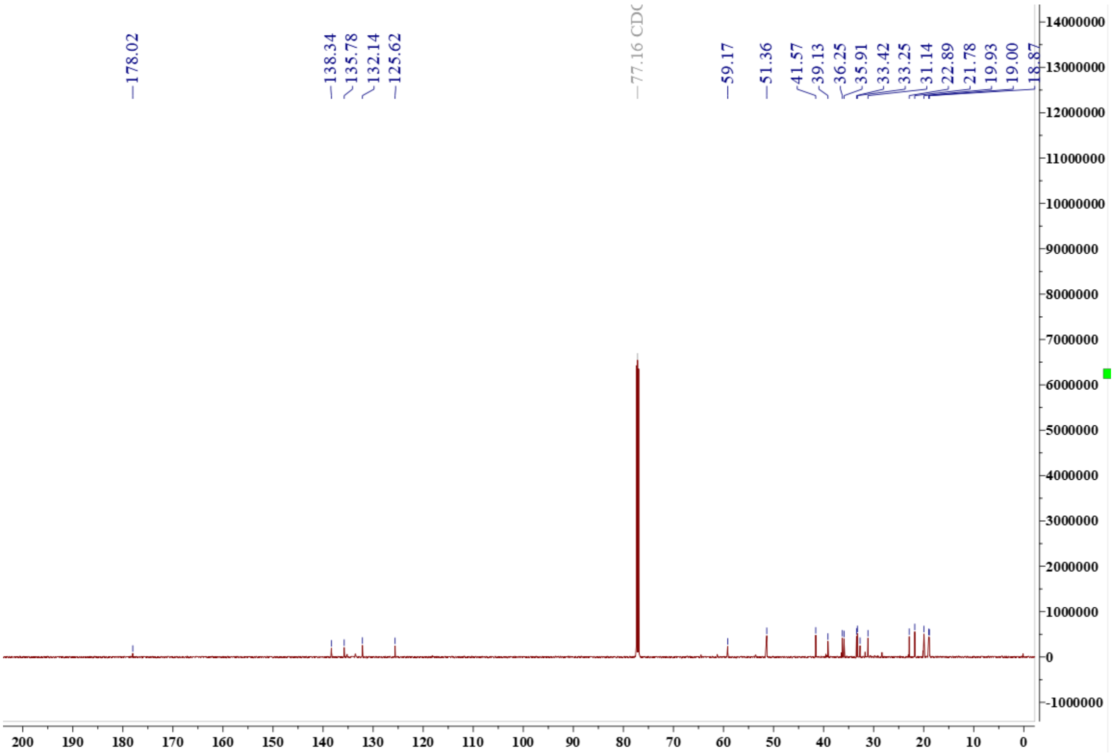

Figure S3. ^{13}C NMR spectrum of compound **1** in CD_3OD

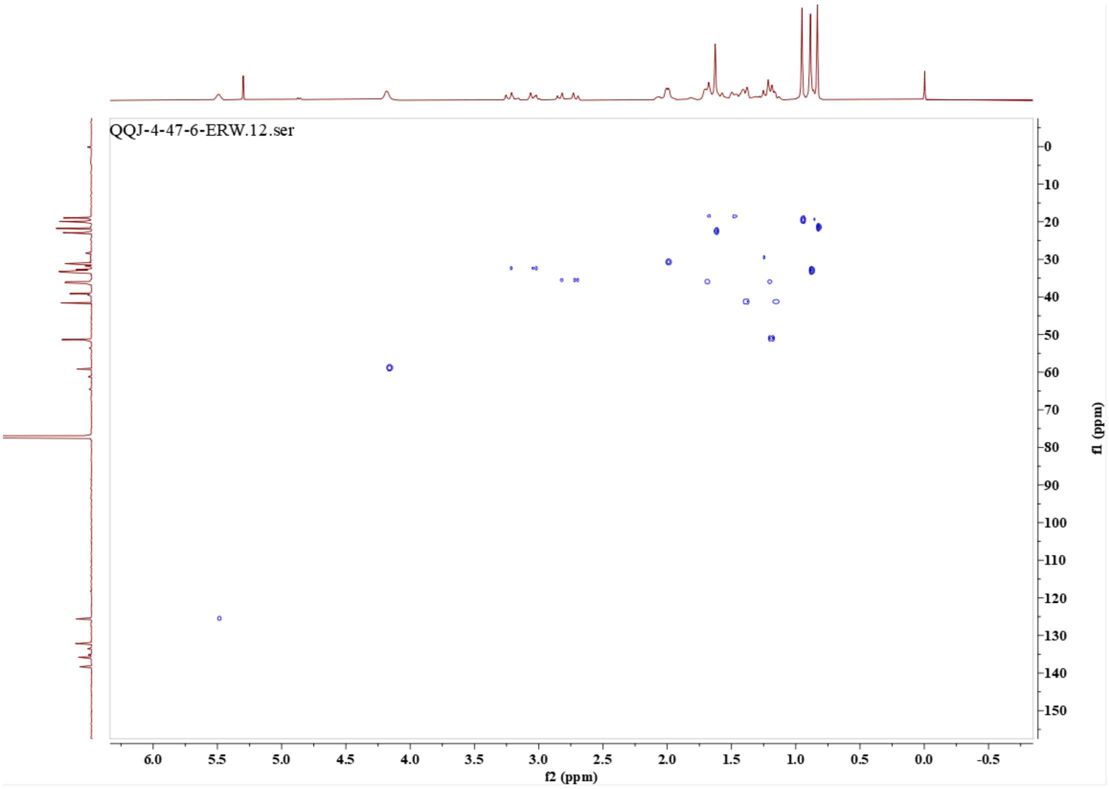

Figure S4. HSQC spectrum of compound **1**

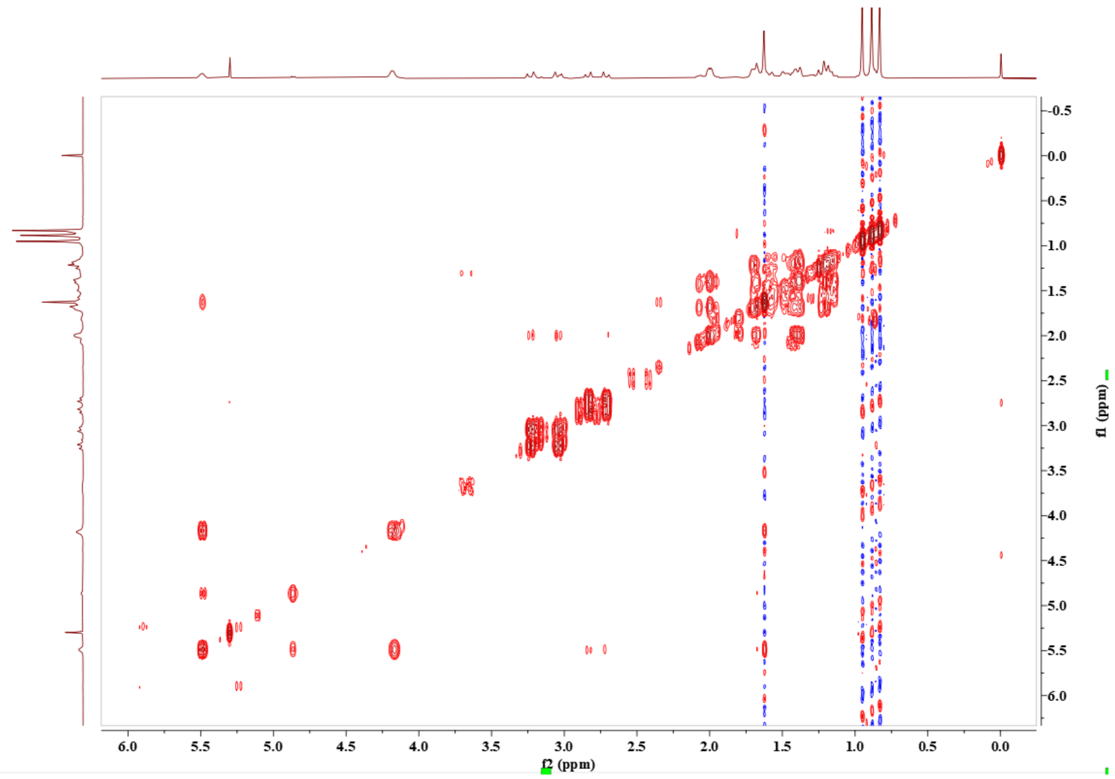

Figure S5. ^1H-^1H COSY spectrum of compound **1**

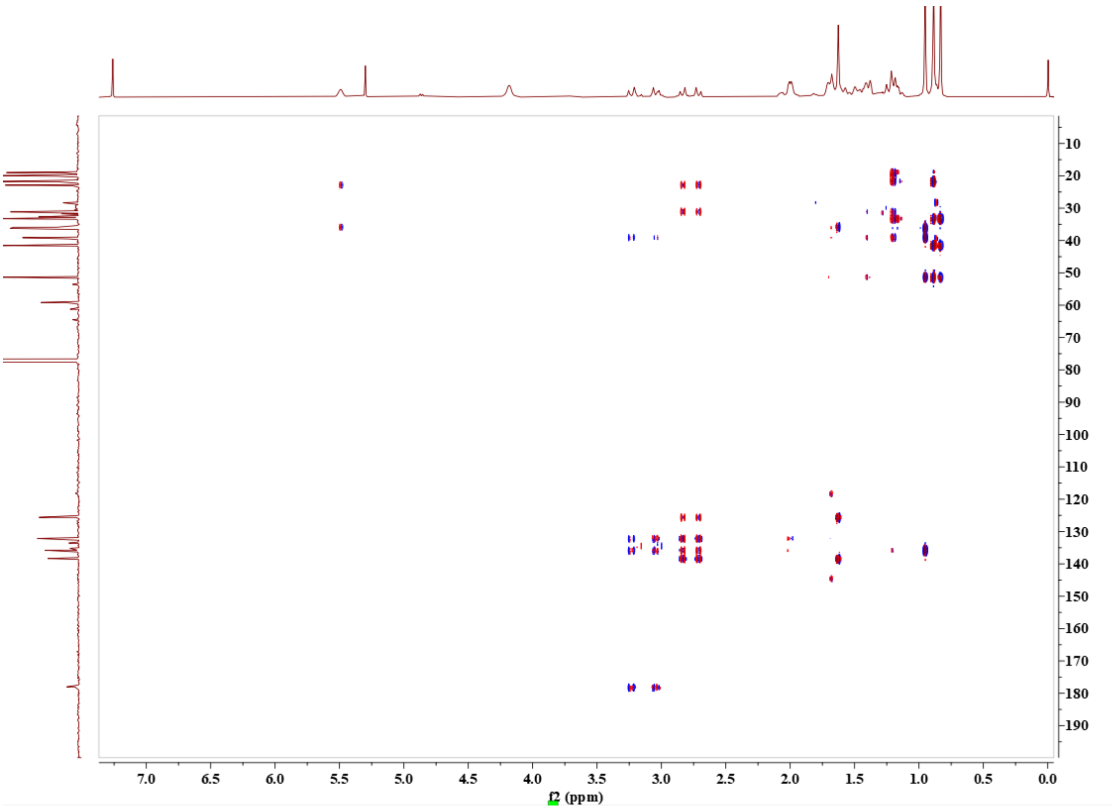

Figure S6. HMBC spectrum of compound **1**

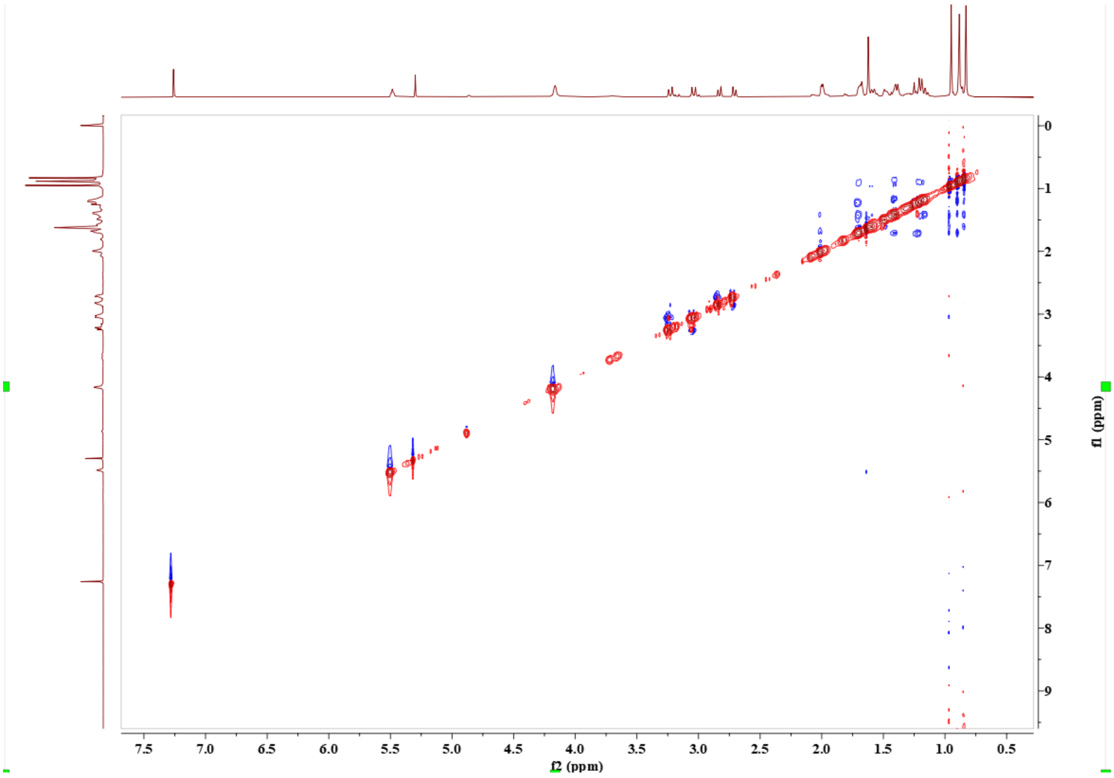

Figure S7. NOESY spectrum of compound **1**

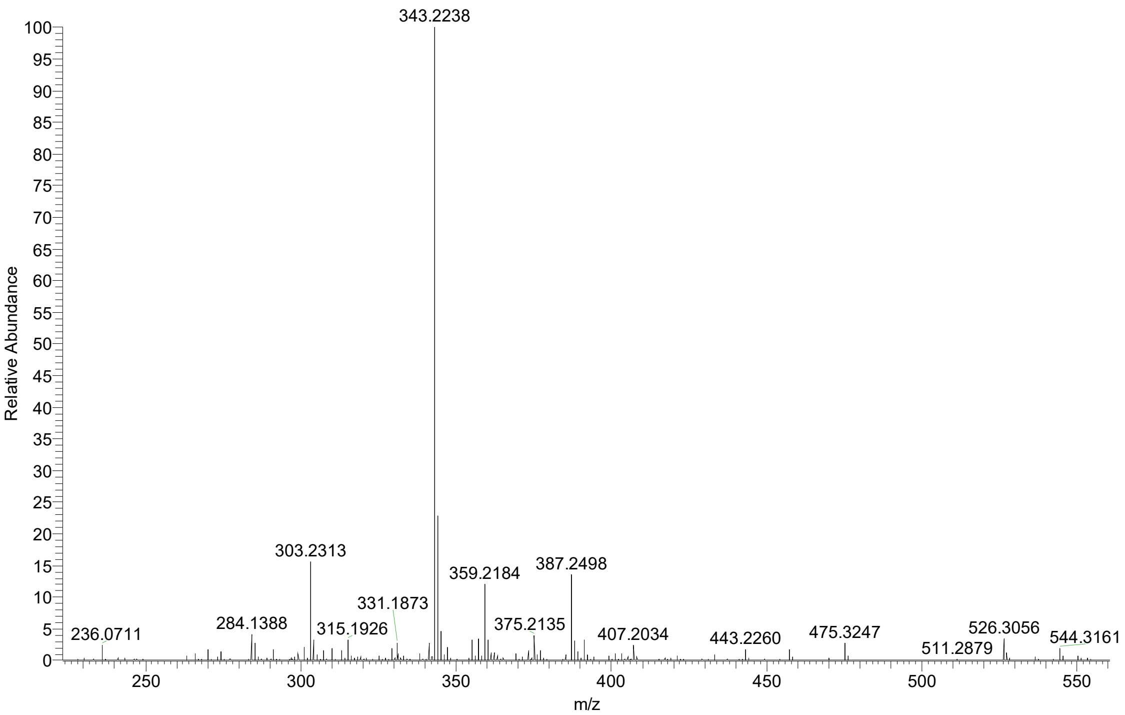

Figure S8. HRESIMS spectrum of compound **2**

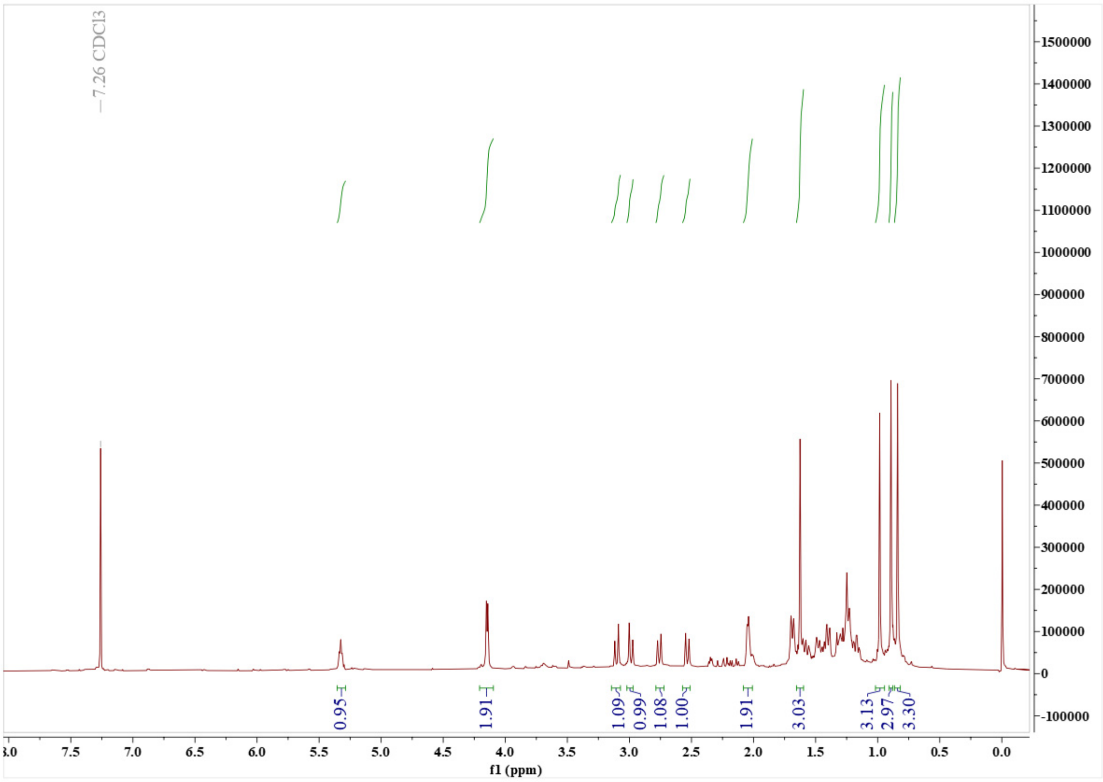

Figure S9. ^{1}H NMR spectrum of compound **2** in $CDCl_3$

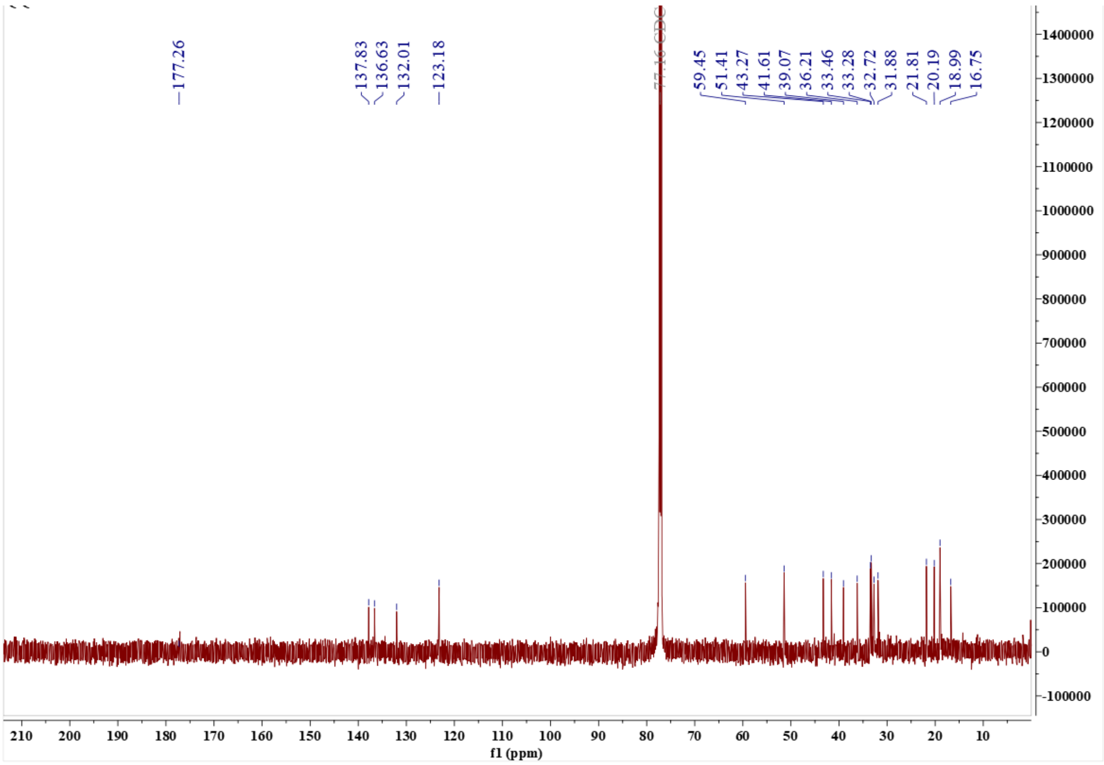

Figure S10. ^{13}C NMR spectrum of compound **2** in $CDCl_3$

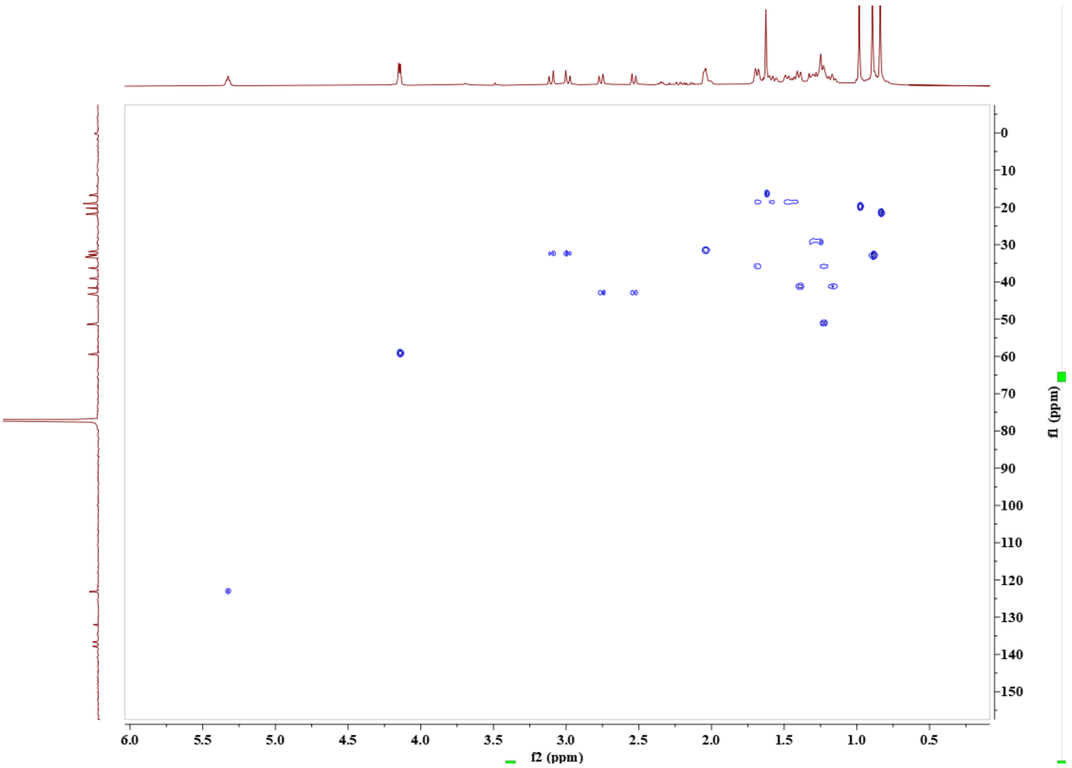

Figure S11. HSQC spectrum of compound **2**

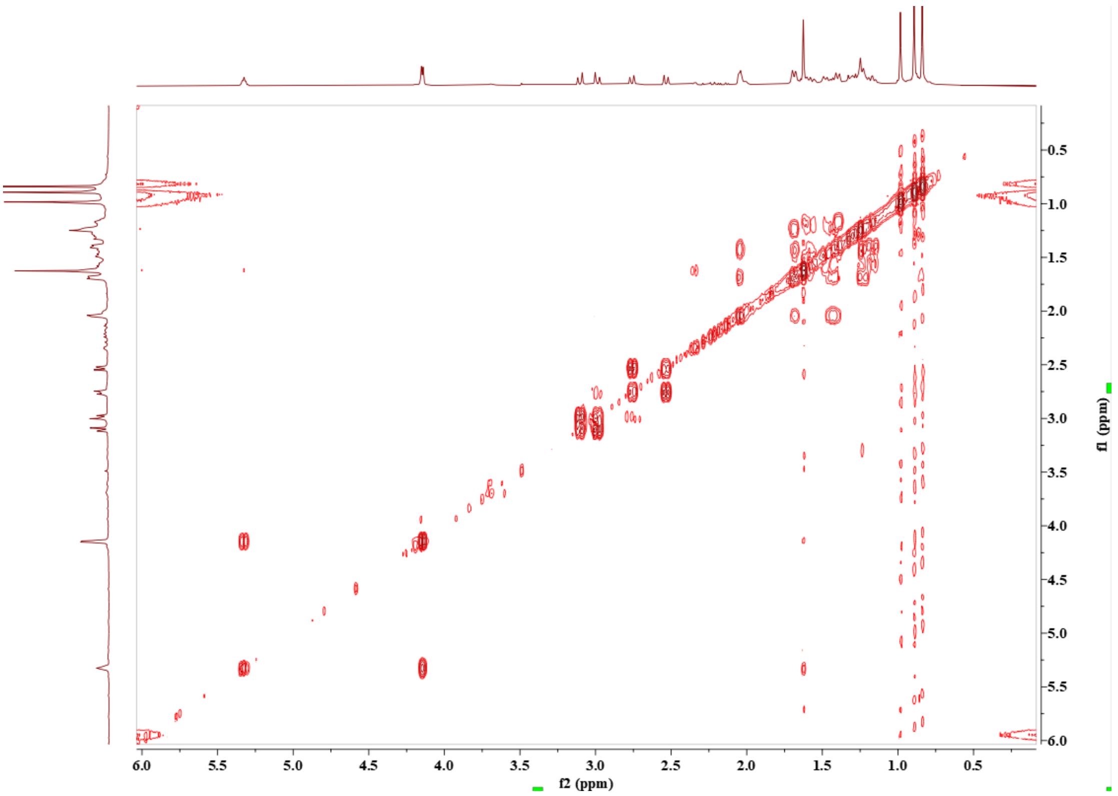

Figure S12. ^{1}H-^{1}H COSY spectrum of compound **2**

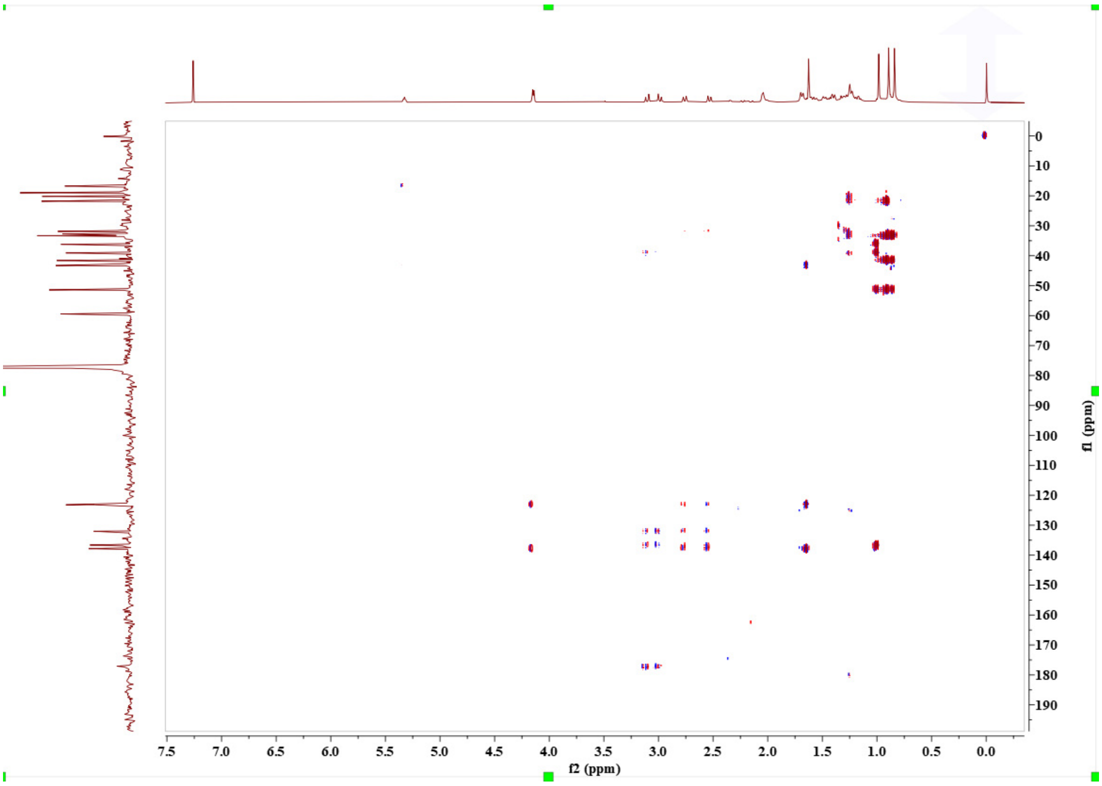

Figure S13. HMBC spectrum of compound **2**

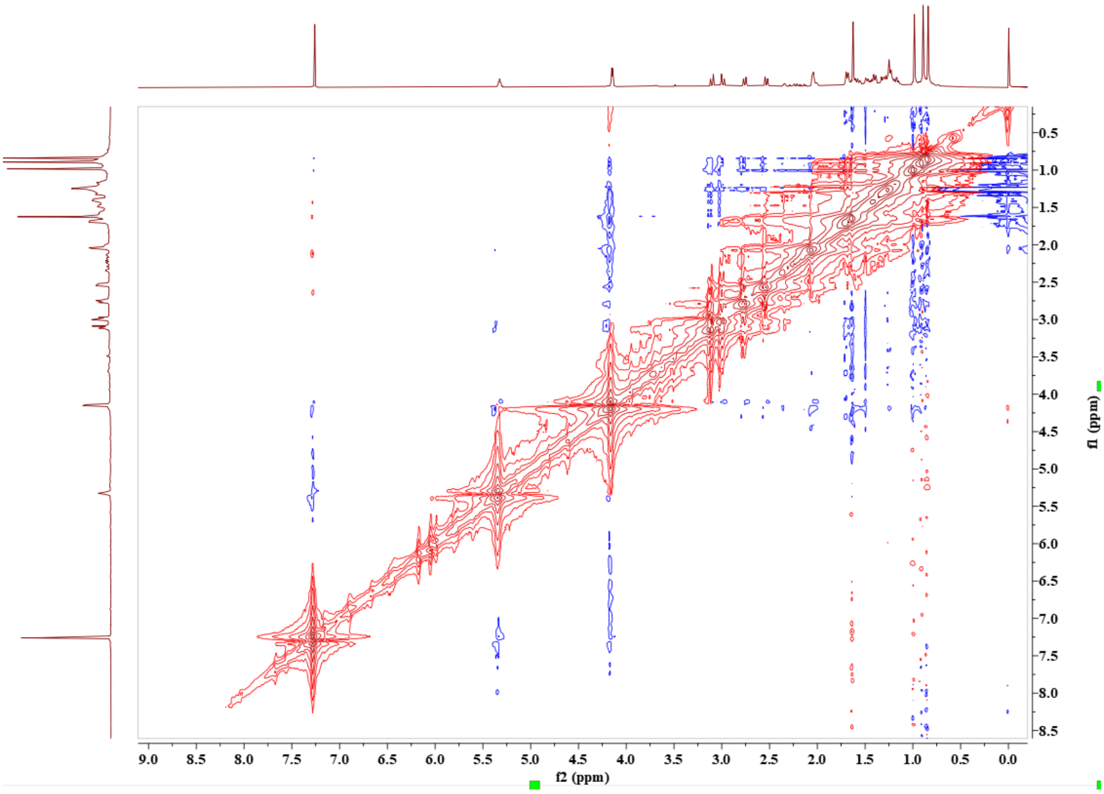

Figure S14. NOESY spectrum of compound **2**

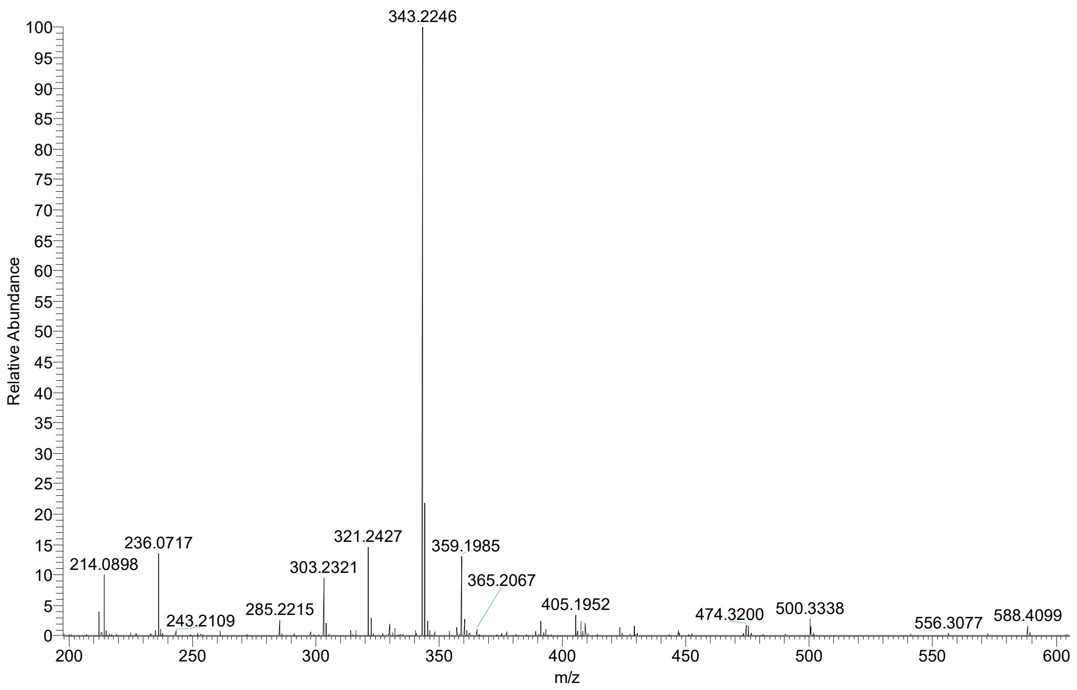

Figure S15. HRESIMS spectrum of compound **3**

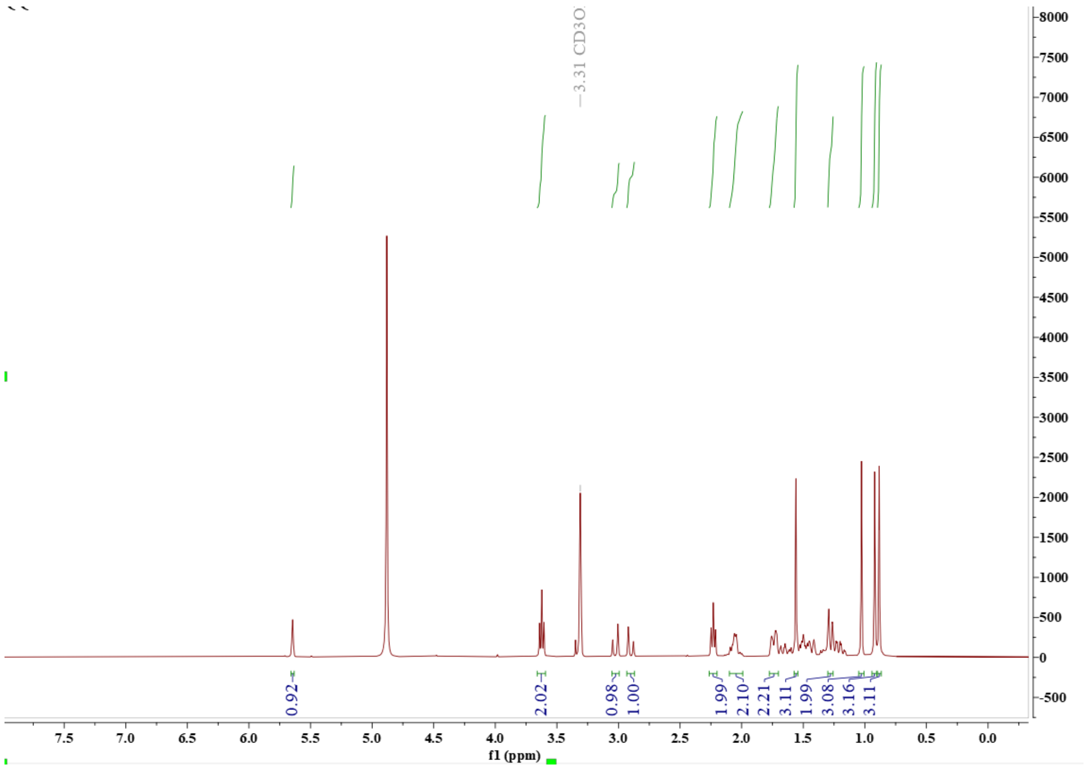

Figure S16. ^{1}H NMR spectrum of compound **3** in CD_3OD

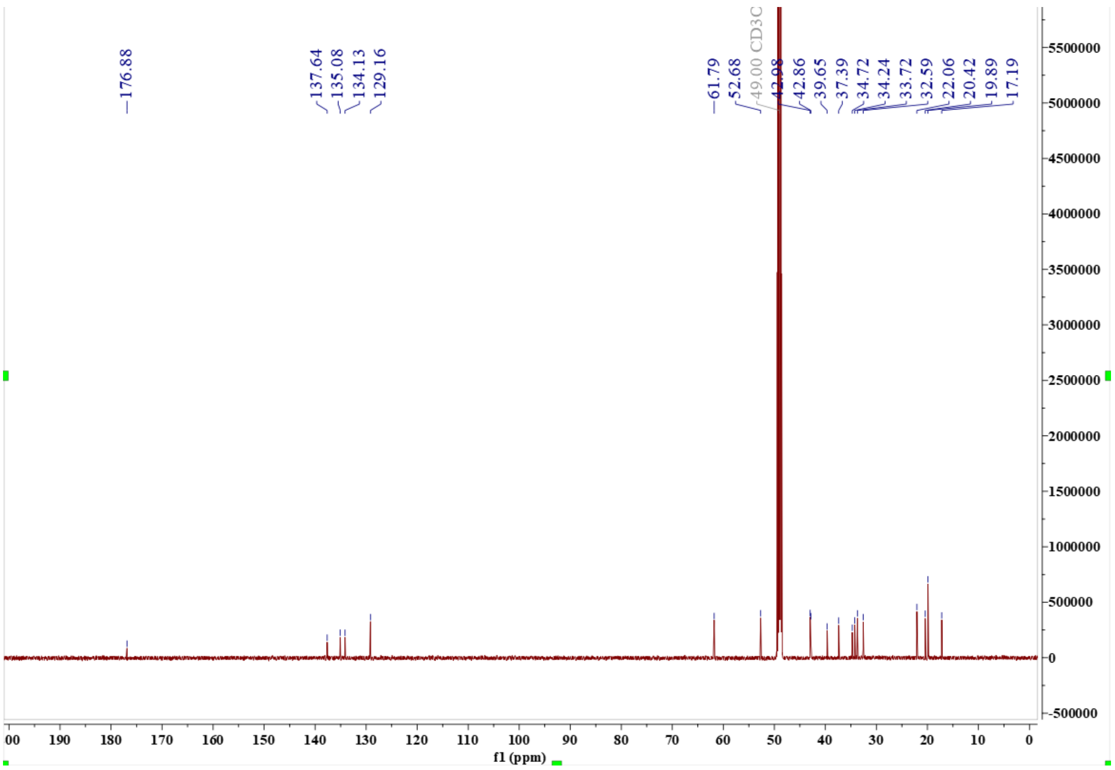

Figure S17. ^{13}C NMR spectrum of compound **3** in CD_3OD

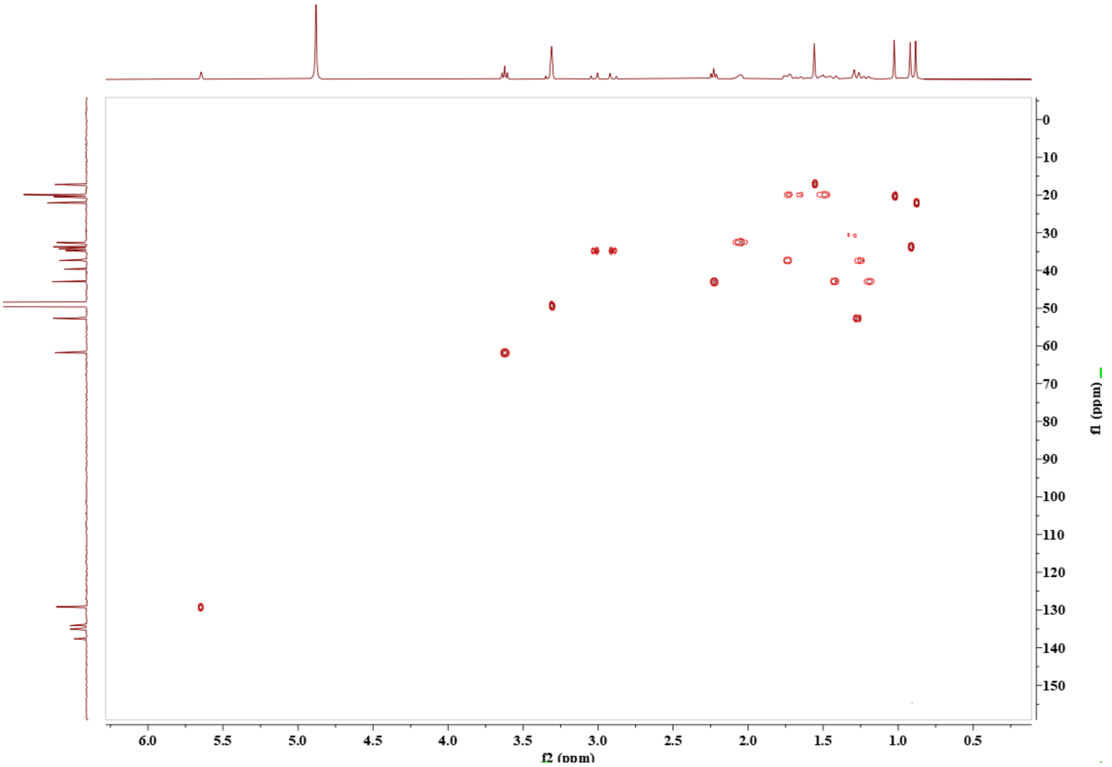

Figure S18. HSQC spectrum of compound **3**

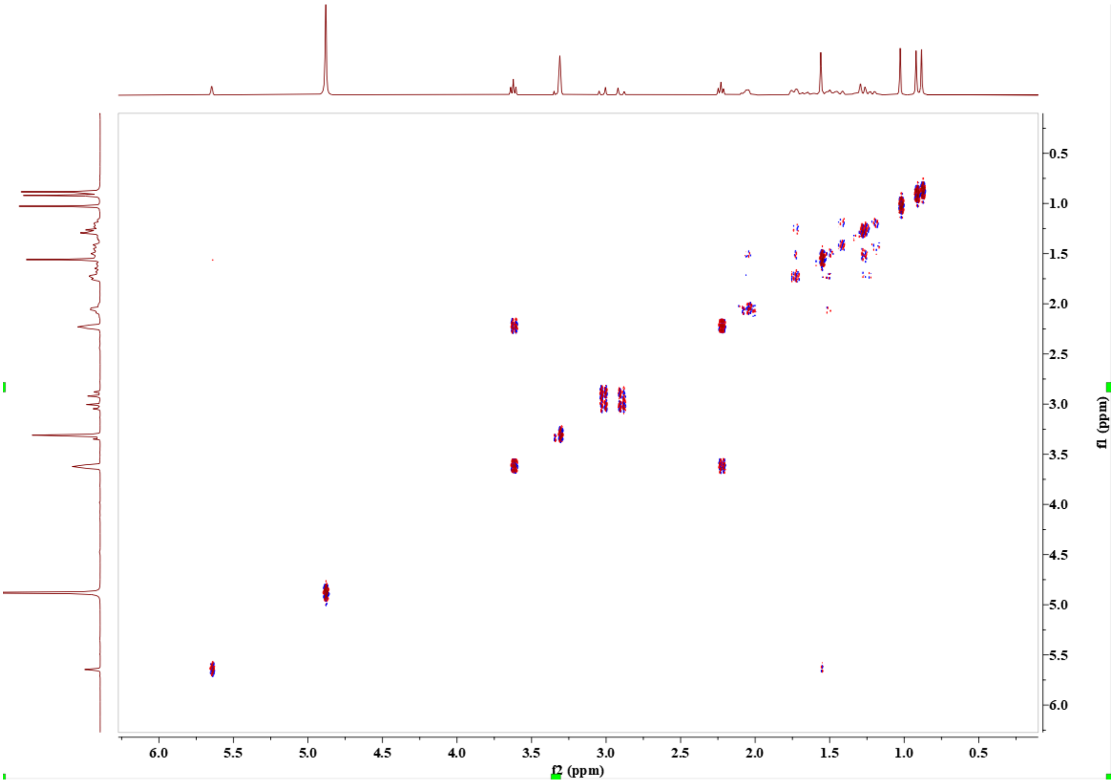

Figure S19. ${}^{1}H$-${}^{1}H$ COSY spectrum of compound **3**

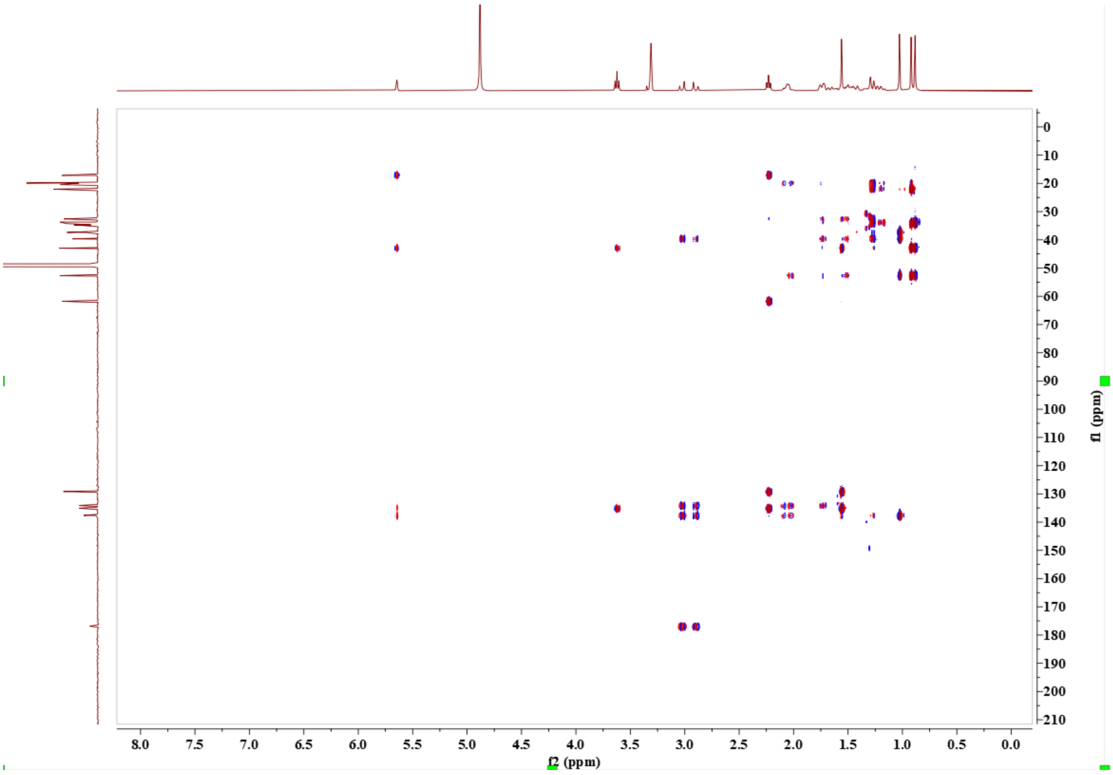

Figure S20. HMBC spectrum of compound **3**.

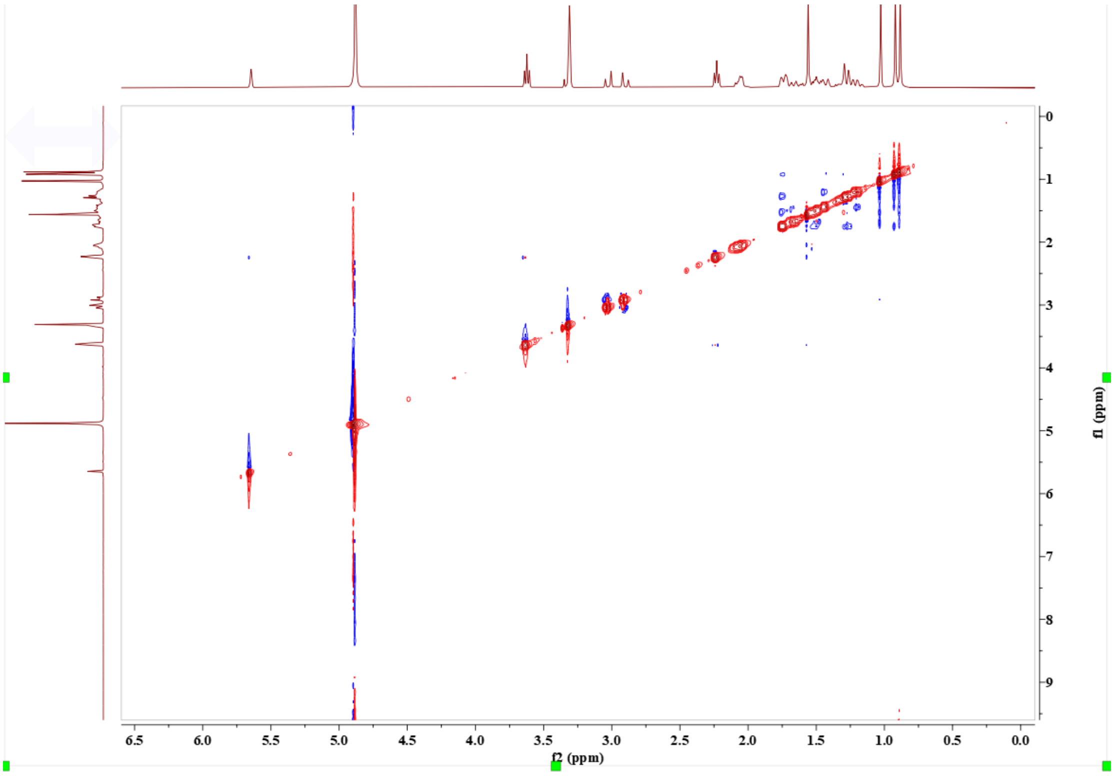

Figure S21. NOESY spectrum of compound **3**

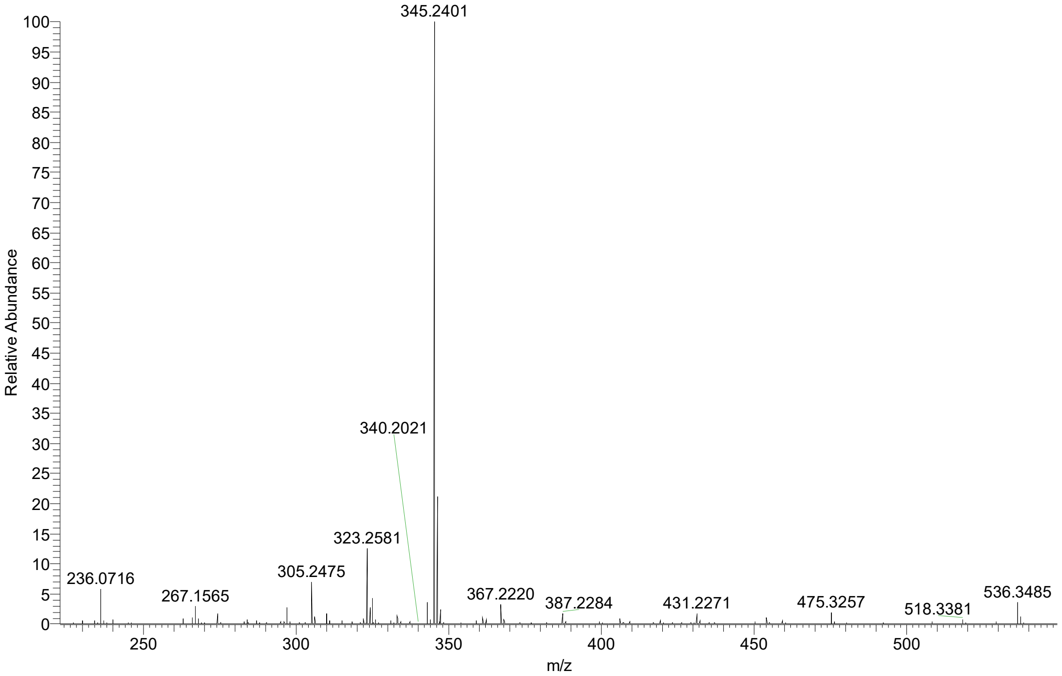

Figure S22. HRESIMS spectrum of compound **4**

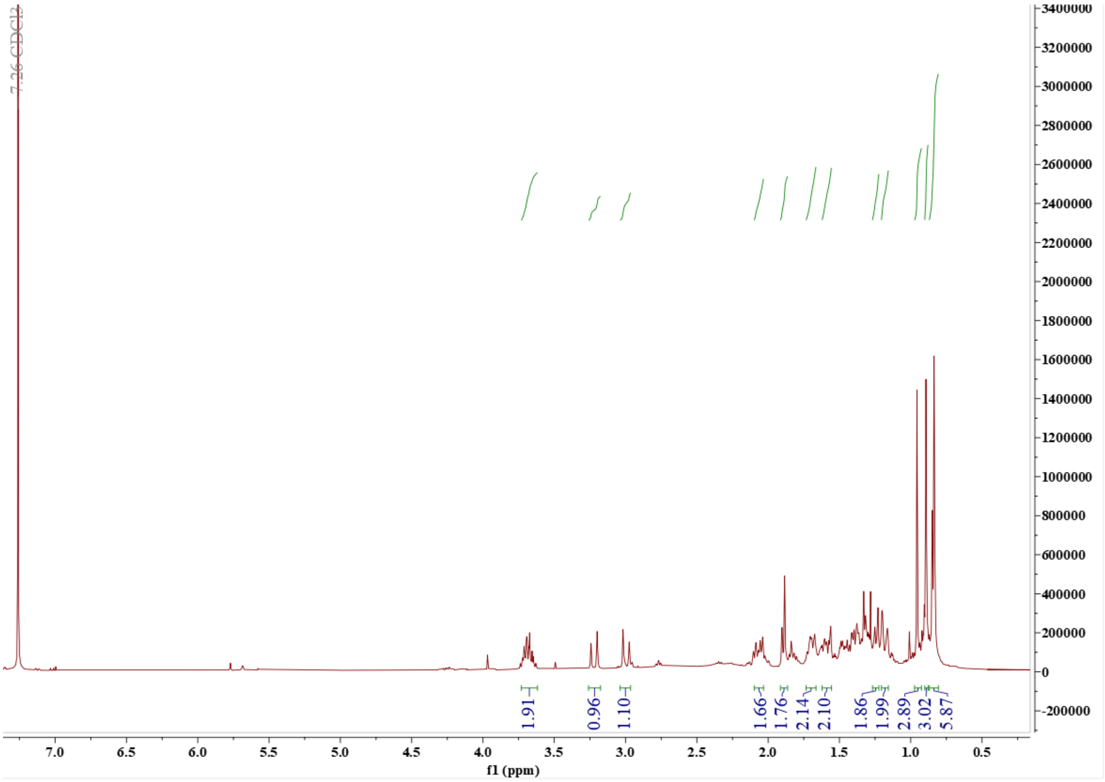

Figure S23. ^{1}H NMR spectrum of compound **4** in $CDCl_3$

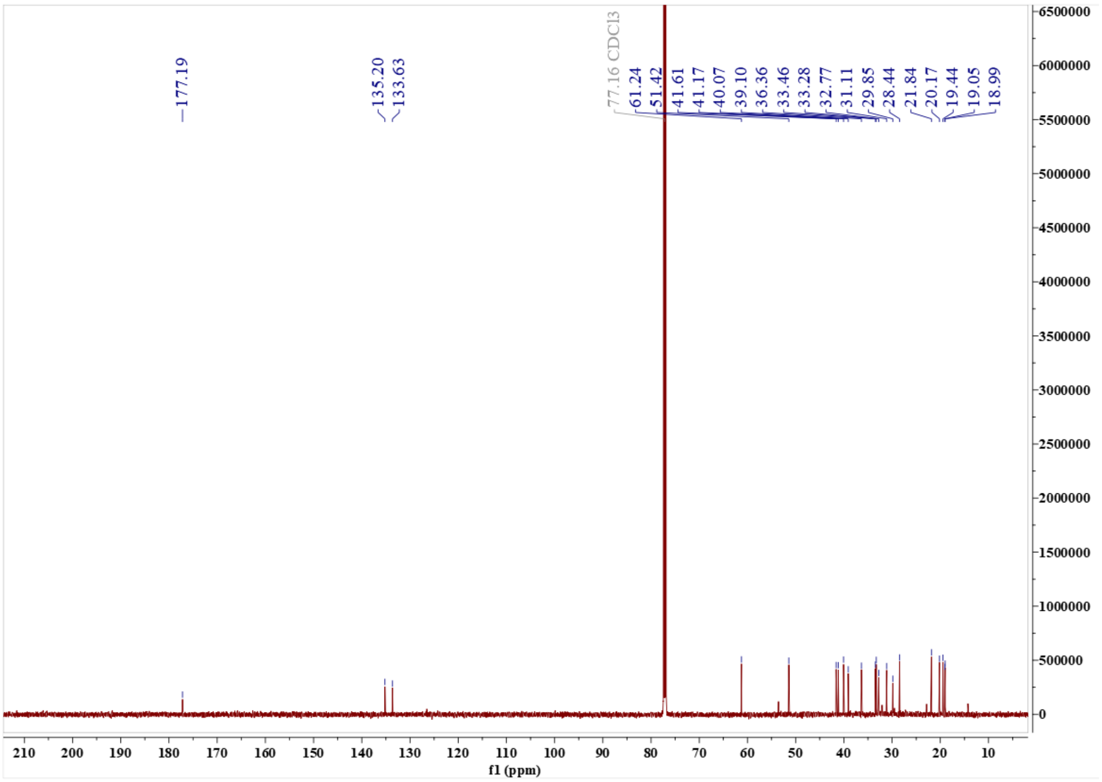

Figure S24. ^{13}C NMR spectrum of compound **4** in $CDCl_3$

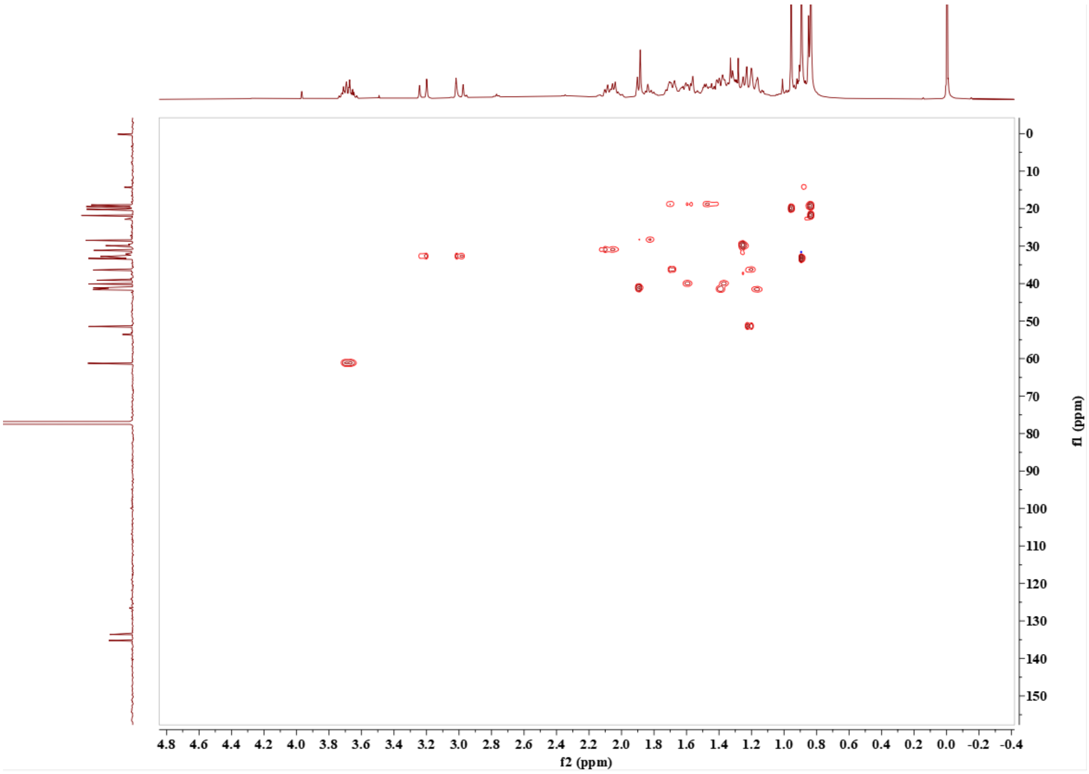

Figure S25. HSQC spectrum of compound **4**

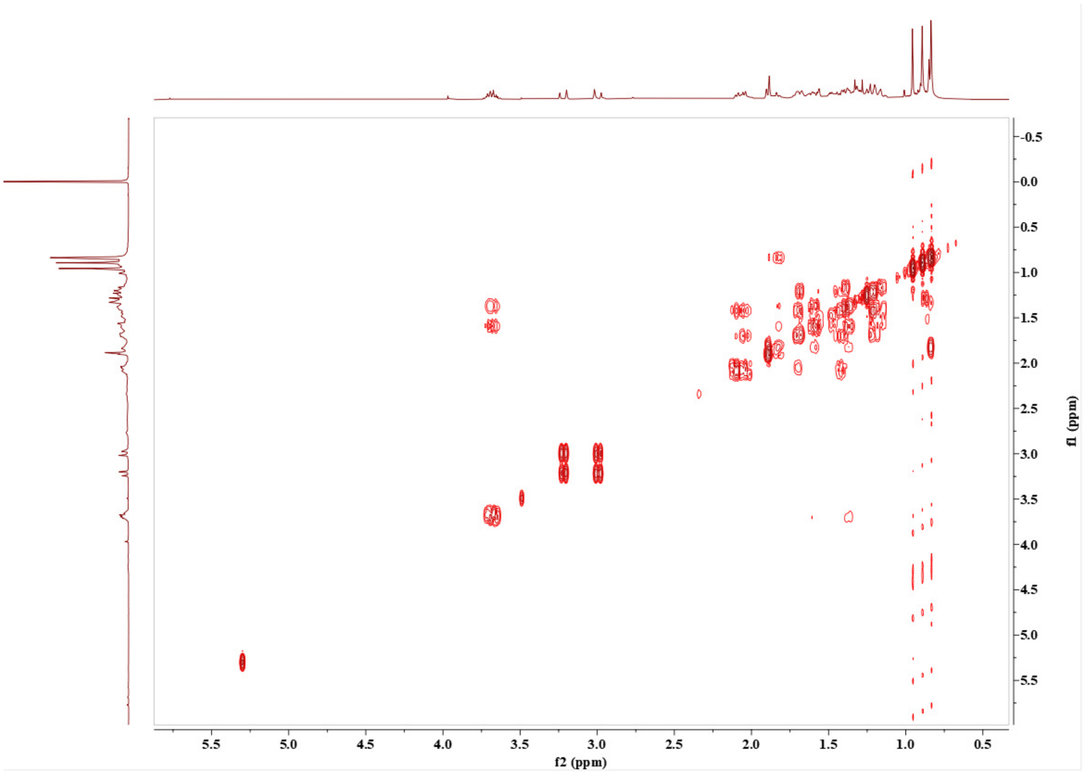

Figure S26. ^{1}H-^{1}H COSY spectrum of compound **4**

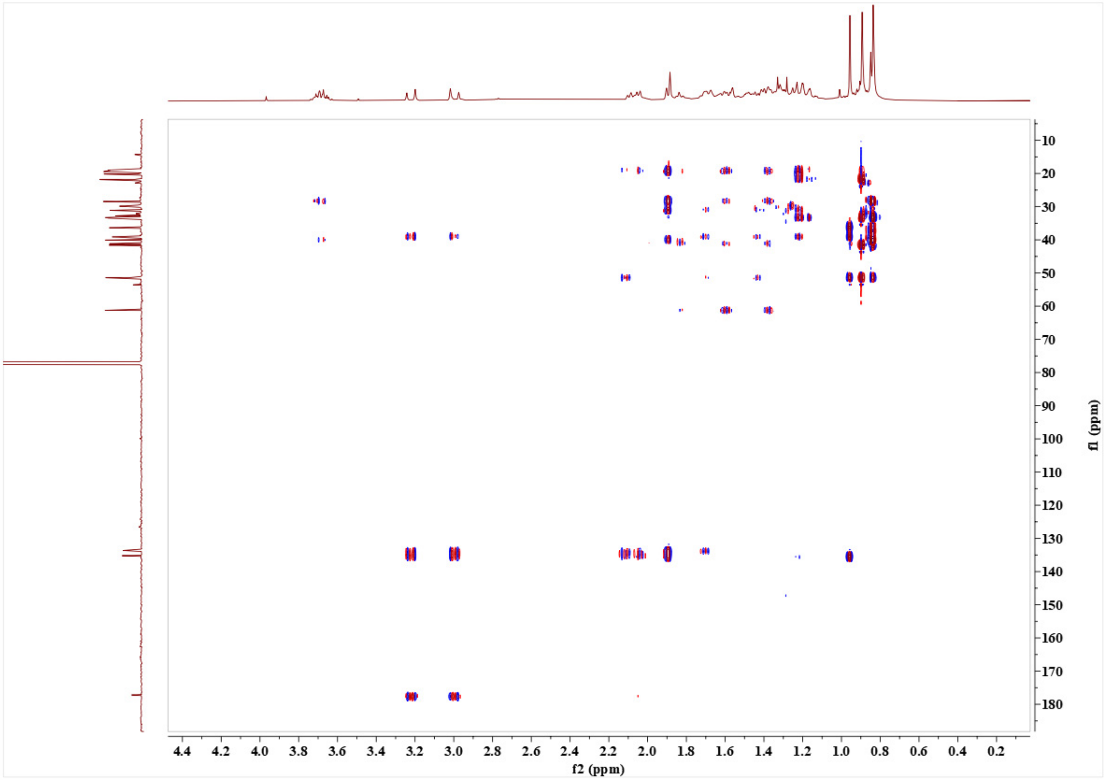

Figure S27. HMBC spectrum of compound **4**

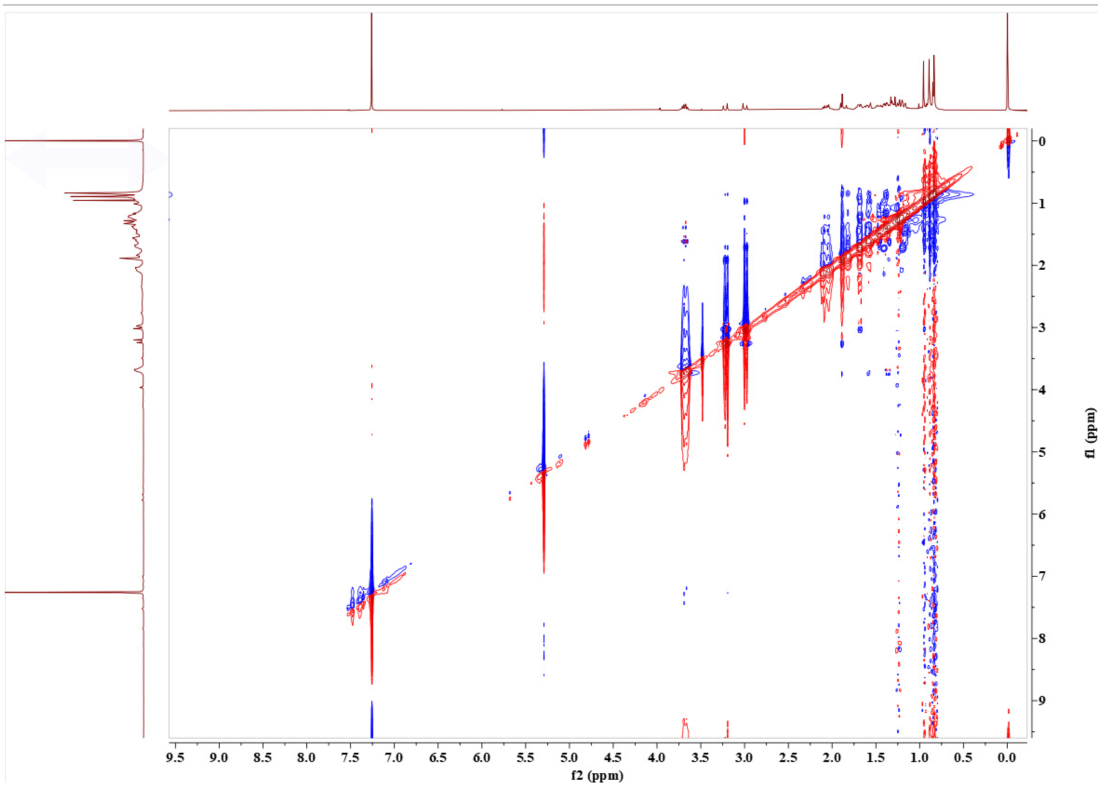

Figure S28. NOESY spectrum of compound **4**

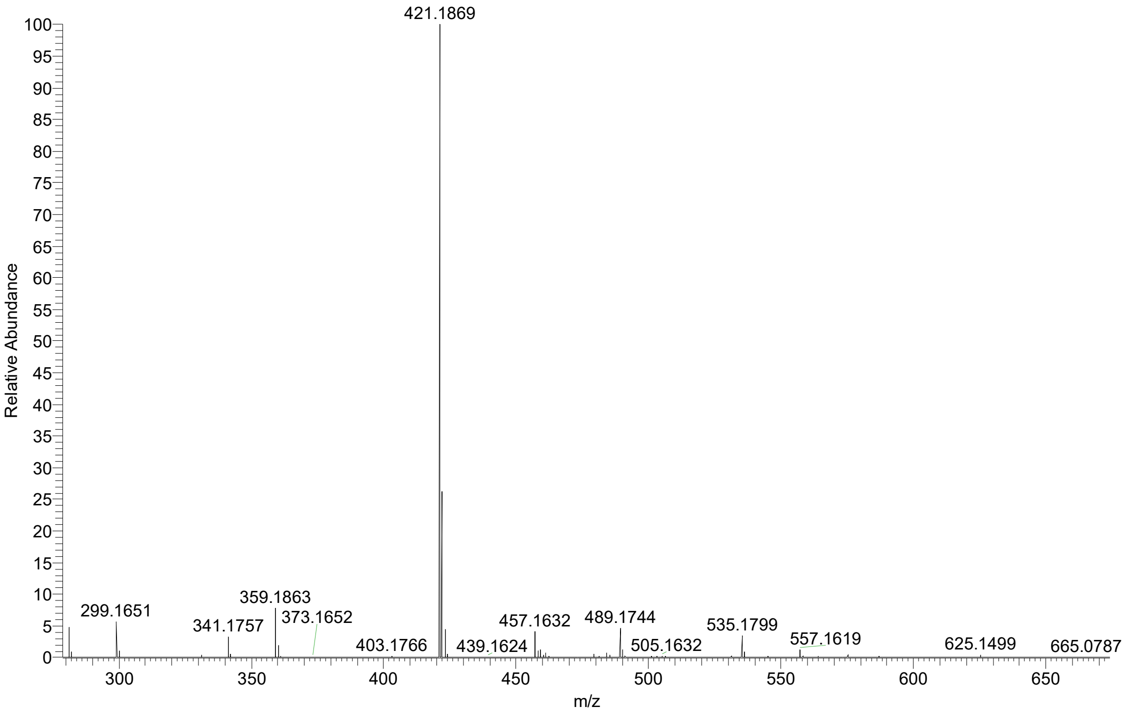

Figure S29. HRESIMS spectrum of compound **5**

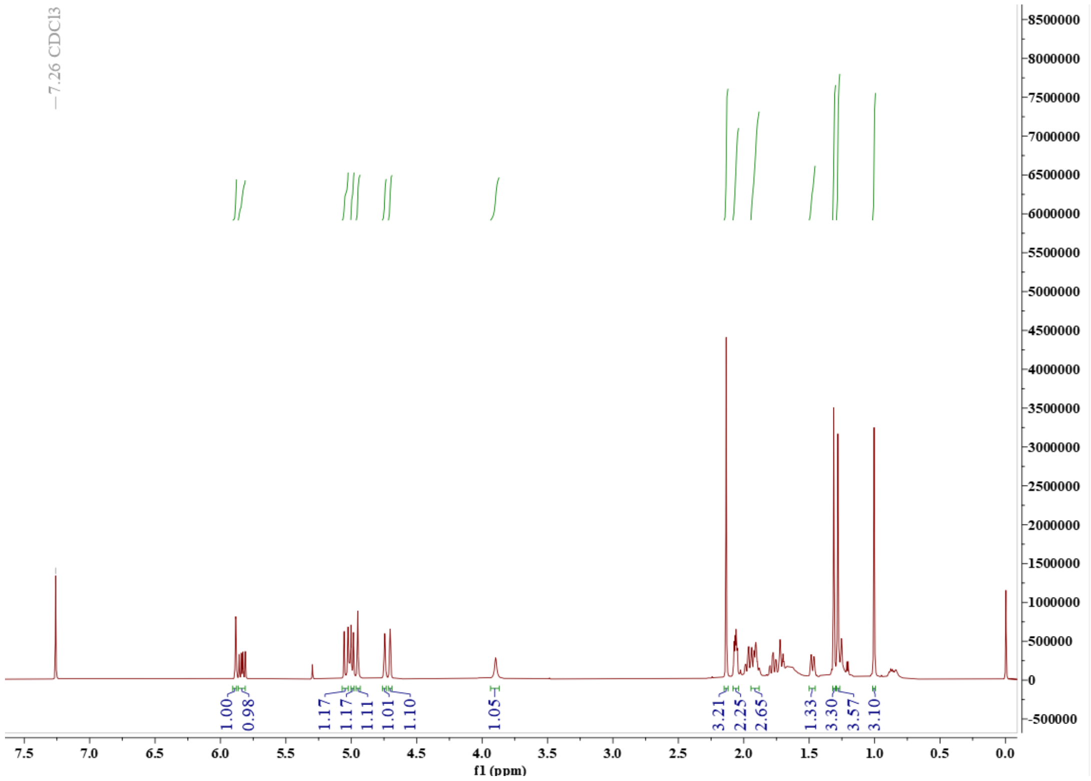

Figure S30. ^{1}H NMR spectrum of compound **5** in $CDCl_3$

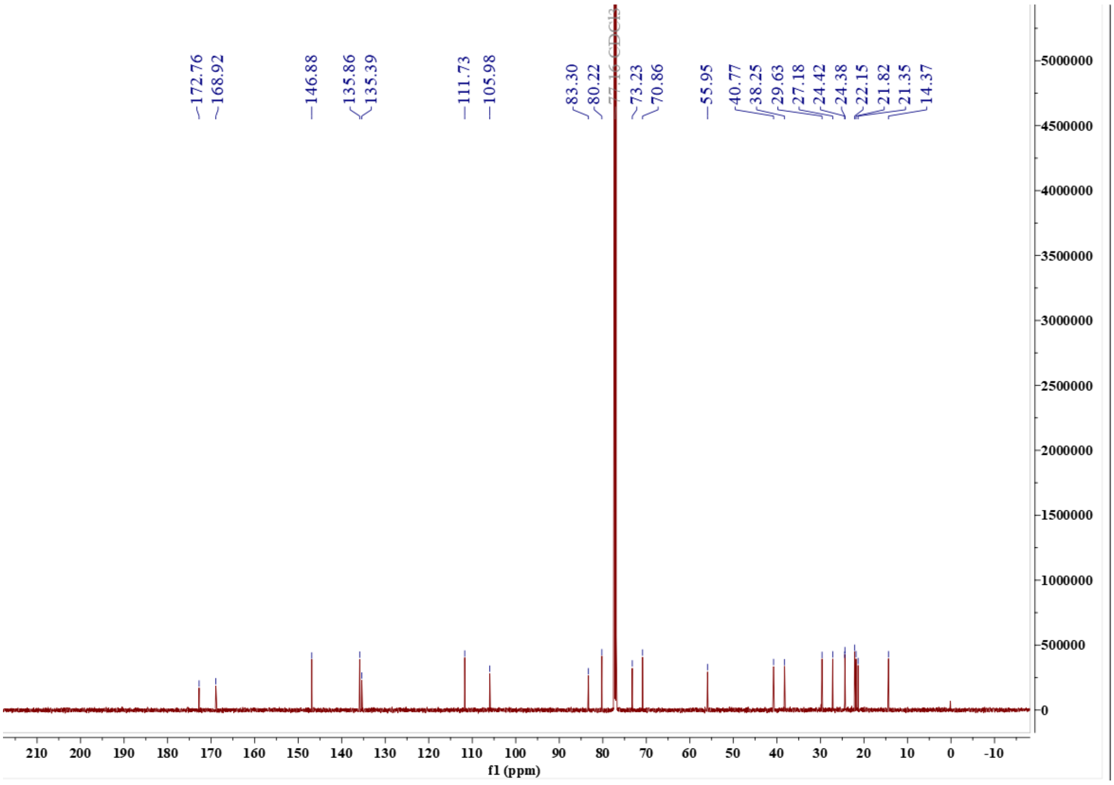

Figure S31. ^{13}C NMR spectrum of compound **5** in $CDCl_3$

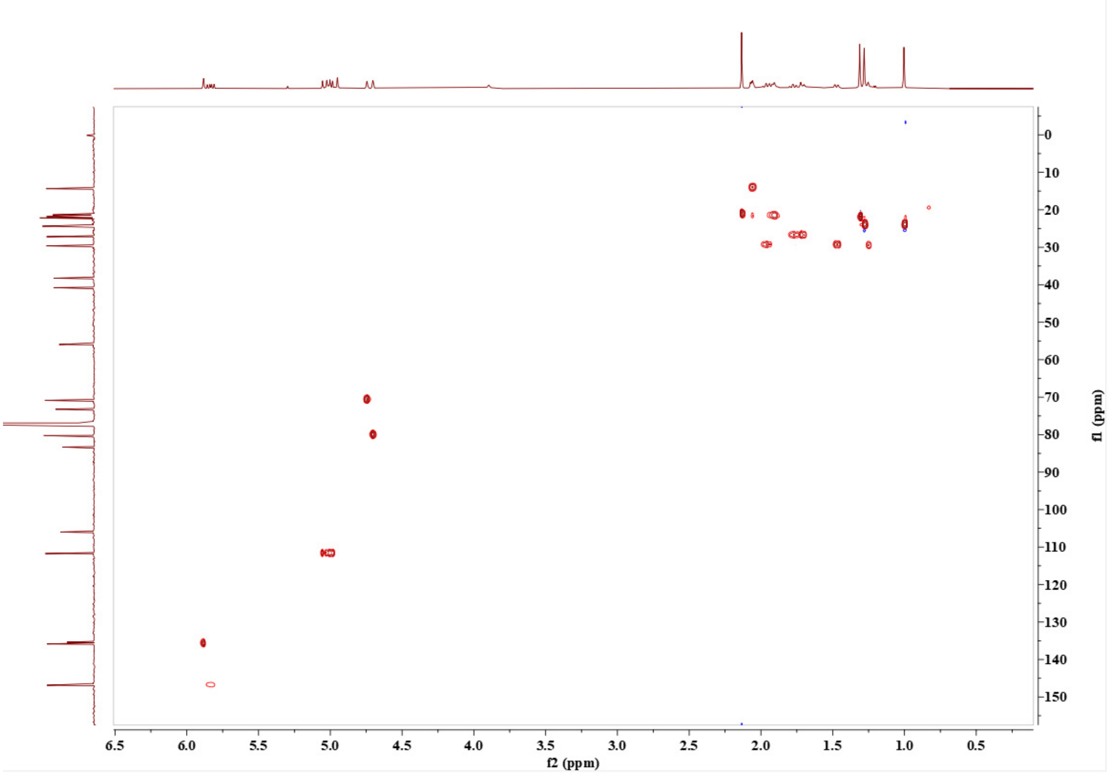

Figure S32. HSQC spectrum of compound **5**

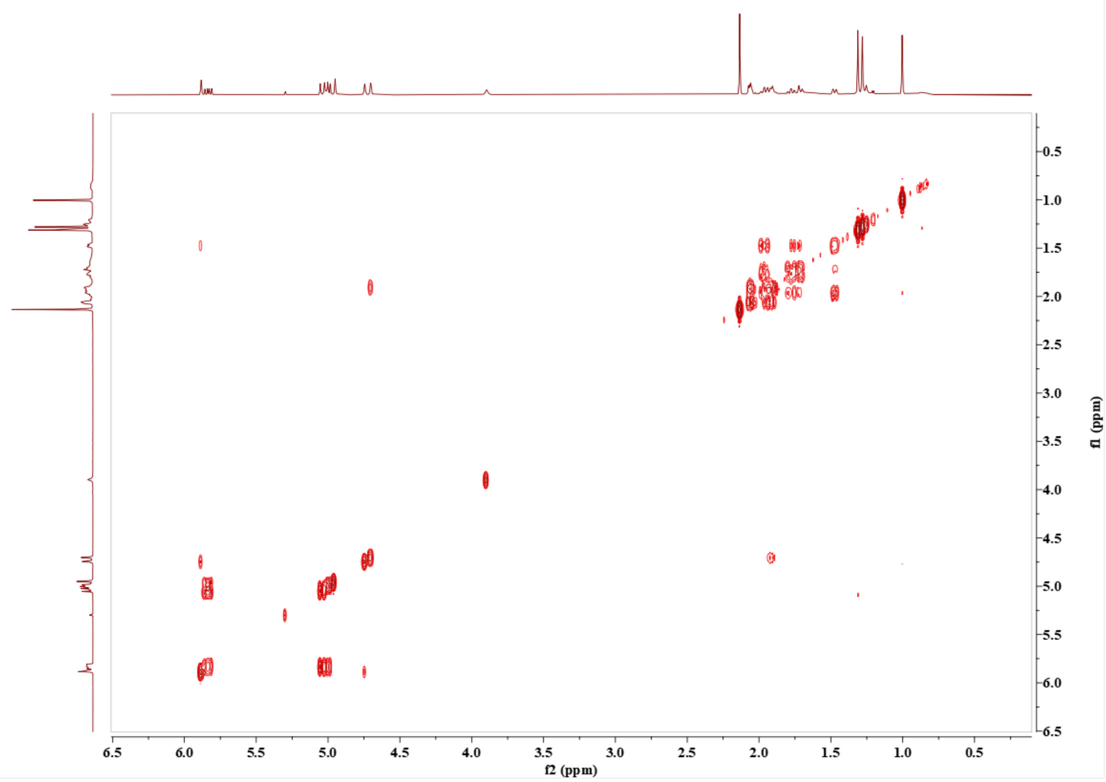

Figure S33. ^{1}H-^{1}H COSY spectrum of compound **5**

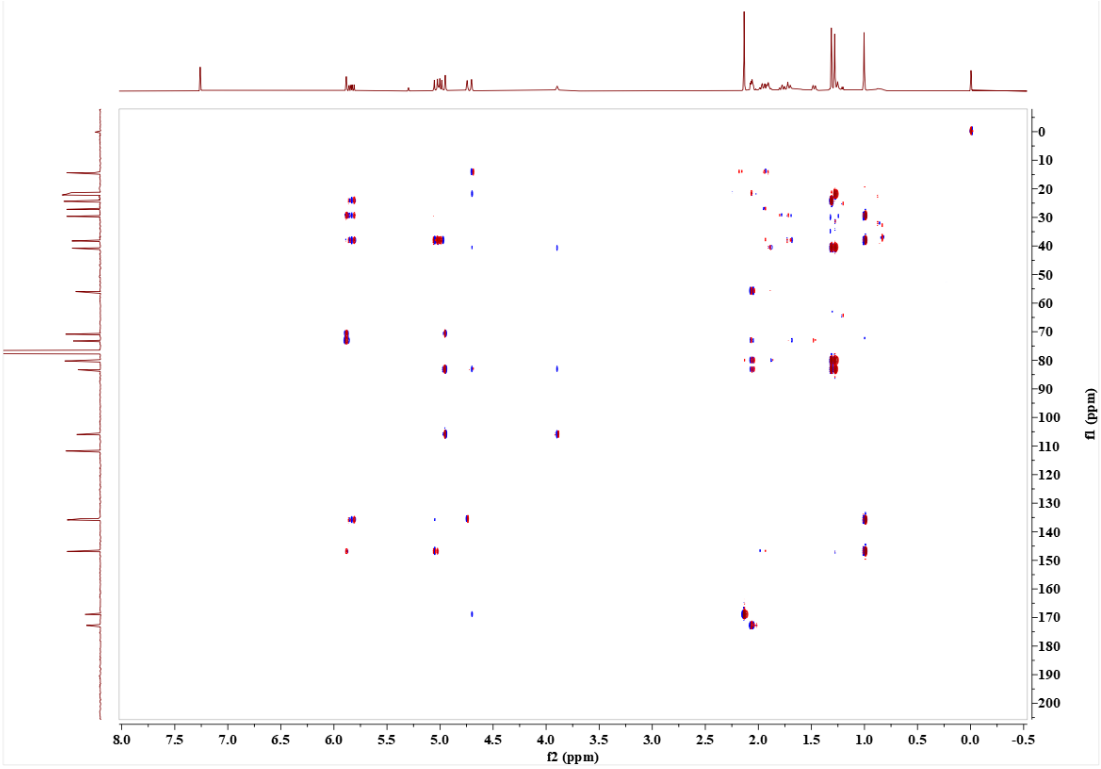

Figure S34. HMBC spectrum of compound **5**

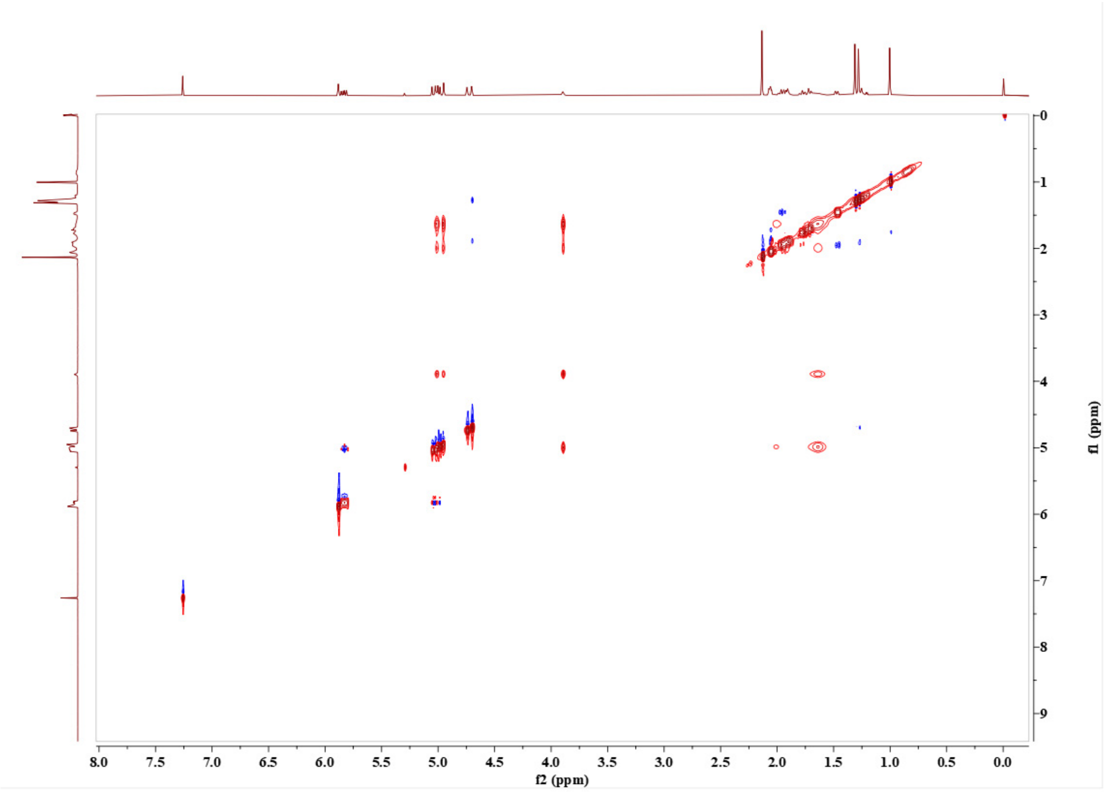

Figure S35. NOESY spectrum of compound **5**

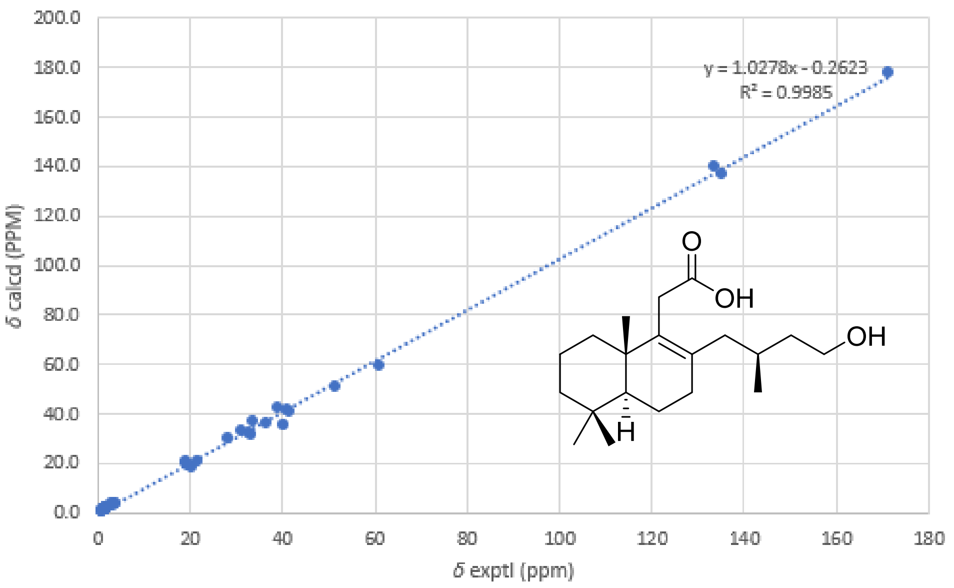

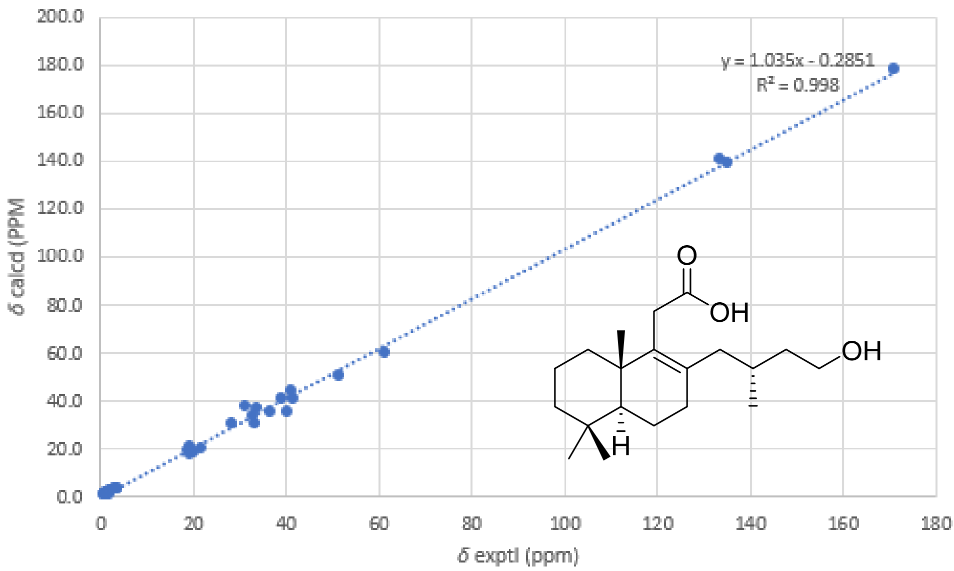

Figure S36. Comparison of the experimental ^{13}C and ^{1}H NMR data of compound **4** and calculated chemical shifts for two potential epimers (5*S*, 10*S*, 14*S* and 5*S*, 10*S*, 14*R*)

Functional	Solvent?		Basis Set		Type of Data	
mPW1PW91	PCM		6-31+G(d,p)		Unscaled Shifts	
	Isomer 1	Isomer 2	Isomer 3	Isomer 4	Isomer 5	Isomer 6
sDP4+ (H data)	98.68%	1.32%	-	-	-	-
sDP4+ (C data)	98.91%	1.09%	-	-	-	-
sDP4+ (all data)	99.99%	0.01%	-	-	-	-
uDP4+ (H data)	99.26%	0.74%	-	-	-	-
uDP4+ (C data)	89.70%	10.30%	-	-	-	-
uDP4+ (all data)	99.91%	0.09%	-	-	-	-
DP4+ (H data)	99.99%	0.01%	-	-	-	-
DP4+ (C data)	99.87%	0.13%	-	-	-	-
DP4+ (all data)	100.00%	0.00%	-	-	-	-

Figure S37. DP4+ analysis of **4** (isomer 1 = 5*S*, 10*S*, 14*S* and isomer 2 = 5*S*, 10*S*, 14*R*)
